# Transcriptome Analysis of the Inhibitory Effect of Sennoside A on the Metastasis of Hepatocellular Carcinoma Cells

**DOI:** 10.3389/fphar.2020.566099

**Published:** 2021-01-12

**Authors:** Jiamei Le, Yi Fu, Qiuqin Han, Yujie Ma, Houlin Ji, Xindong Wei, Yifan Chen, Yongning Sun, Yueqiu Gao, Hailong Wu

**Affiliations:** ^1^ Shanghai University of Medicine & Health Sciences Affiliated Zhoupu Hospital, Shanghai, China; ^2^ Shanghai Key Laboratory of Molecular Imaging, Collaborative Innovation Center for Biomedicine, Shanghai University of Medicine & Health Sciences, Shanghai, China; ^3^ Department of Traditional Chinese Medicine, Shanghai Jiao Tong University Affiliated Sixth People’s Hospital, Shanghai, China; ^4^ Department of Cardiology, Shanghai Municipal Hospital of Traditional Chinese Medicine, Shanghai University of Traditional Chinese Medicine, Shanghai, China; ^5^ Institute of Clinical Immunology, Department of Liver Diseases, Central Laboratory, ShuGuang Hospital Affiliated to Shanghai University of Traditional Chinese Medicine, Shanghai, China; ^6^ Laboratory of Cellular Immunity, Shuguang Hospital, Shanghai University of Traditional Chinese Medicine, Shanghai, China

**Keywords:** Sennoside A, hepatocellular carcinoma, transcriptome analysis, migration, invasion, metastasis

## Abstract

Sennoside A (SA) is a bioactive component of *Rheum officinale Baill.* with an activity of irritant laxative, which has been reported to possess therapeutic potential in various diseases or conditions including obesity, insulin resistance, liver steatosis, prostate cancer and pancreatic cancer progression. However, whether SA has therapeutic potential in hepatocellular carcinoma (HCC) treatment remains elusive. In this study, we treated two HCC cell lines, HepG2 and SMMC-7721 with SA and found that SA selectively inhibited the growth of HCC cells by proliferation assay. SA has a good inhibitory effect on proliferation of HepG2 cells in a concentration dependent manner, but there was no effect on SMMC-7721 cells. Then we conducted transwell assays and transcriptome analysis in HCC cells and examined the effects of SA on HCC *in vivo*. The results showed that SA significantly inhibited the migration and invasion of HCC. Comparison of RNA-seq transcriptome profiles from control groups and SA-treated groups identified 171 and 264 differentially expressed genes (DEGs) in HepG2 and SMMC-7721 cells respectively, in which includes 2 overlapping up-regulated DEGs and 12 overlapping down-regulated DEGs between HepG2 and SMMC-7721 cells. The qPCR were applied to investigate the transcriptional level of 9 overlapping down-regulated DEGs related to cancer metastasis, and the results were consistent with RNA-seq data. The dominate pathways including Wnt signaling pathway, TNF signaling pathway, VEGF signaling pathway, and NF-κB signaling pathway were strongly inhibited by SA, which are involved in regulating cancer metastasis. Finally, we confirmed that the downregulation of *KRT7* and *KRT81* could inhibit HCC metastasis. This study has provided new insight into the understanding of the inhibitory effects and potential targets of SA on the metastasis of HCC.

## Introduction

Primary liver cancer was the sixth most commonly occurring cancer and the fourth leading cause of cancer death worldwide in 2018 (Bray et al., 2018). Hepatocellular carcinoma (HCC) is the most common type of primary liver cancer in most countries, accounting for approximately 75% of total cases (Altekruse et al., 2011). There are multimodal therapies used to treat HCC in the past several decades, including surgical resection, chemotherapy, local radiotherapy, immune and systemic treatment (Hartke et al., 2017). However, the therapeutic outcomes of HCC are still unsatisfactory due to post-surgical recurrence and treatment resistance. Moreover, the rate of post-surgical recurrence and metastasis in HCC is extremely high (Xia et al., 2013). Therefore, clarifying the underlying mechanisms of HCC progression may provide new strategies for the diagnosis and treatment of this deadly disease.

Recently, many medicinal plants have been investigated as an alternative of chemotherapeutic drug to treat cancer for their therapeutic efficacy and safety. *Rheum officinale Baill.* (Da Huang) is one of the oldest and well-known Traditional Chinese Medicines (TCM) that has been widely used for the treatment of constipation, jaundice, gastro-intestinal haemorrhage, and ulcers (Li et al., 2009). Currently, many TCM preparations containing Da Huang, have been clinically used for treating liver diseases, inflammation and cancer (Li et al., 2007; Li et al., 2009). Moreover, a large number of studies *in vitro* and *in vivo* have reported that Da Huang, TCM preparations containing Da Huang, the Da Huang extracts and the major anthraquinone compounds derived from Da Huang (such as emodin, rhein, aloe-emodin) have therapeutic potential in the treatment of hepatocellular carcinoma (Kupchan and Karim, 1976; Ha et al., 2004; Cao et al., 2005; Cha et al., 2005; Kim et al., 2005; Huang et al., 2007; Lu et al., 2007; Shi et al., 2008; Lin et al., 2009; Tsai et al., 2013; Lin et al., 2016; Lee et al., 2017; Tan et al., 2019).

Sennoside A (SA), a major anthraquinone active constituent of *Rheum officinale Baill.*, is widely used as irritant laxative, weight-loss herbal medicines or dietary supplements in China and other Asian countries. It has been reported that SA has antibacterial effect and inhibitory effect on the α-glucoamylase enzyme activity and HIV-1 replication (Choi et al., 2006; Sharma et al., 2012; Esposito et al., 2016). Our previous studies have demonstrated that SA may restore the function of microbiota–GLP1 axis to improve glucose metabolism in the obese mice (Le et al., 2019), and could improve the liver steatosis in non-alcoholic fatty liver disease (NAFLD) mice (Le et al., 2018). Furthermore, a recent study has identified SA as a novel inhibitor of the slingshot family proteins to inhibit metastasis in pancreatic cancer (Lee et al., 2017). It is also reported that SA can promote the autophagic death of prostate cancer cell line LNCap by inhibiting JAK2/STAT3 signaling transduction and up-regulating levels of autophagy and apoptotic proteins (Wu et al., 2019). Notwithstanding these studies, the biological effect of SA on cancer is still poorly understood and its effect on HCC has not been reported.

In this study, we investigate the role of SA on HCC growth and metastasis *in vitro* and *in vivo*, and examine the transcriptome changes in HCC cells in response to SA treatment. To the best of our knowledge, this study is the first functional and transcriptome analyses of SA on HCC cells. The results will contribute to the identification of target genes of SA and provide a more comprehensive understanding of the role of SA in suppressing cancer metastasis in HCCs.

## Materials and Methods

### Chemicals and Reagents

Sennoside A (98% in purity) was purchased from Selleck Chemicals (Shanghai, China). Dulbeccoʼs modified Eagleʼs medium (DMEM), fetal bovine serum (FBS), penicillin, and streptomycin were purchased from Gibco (Carlsbad, CA, USA). Other chemicals were purchased from Sigma-Aldrich Co. Ltd. (Shanghai, China) unless stated otherwise.

### Cell Lines and Cell Culture

Human HCC cell lines HepG2 and SMMC-7721 were acquired from the Cell Bank of Type Culture Collection of the Chinese Academy of Sciences (Shanghai, China) and cultured in DMEM containing 10% FBS, 100 U/ml penicillin, and 100 μg/ml streptomycin at 37°C in culture chamber with 5% CO_2_. Cell culture medium was refreshed every other day.

### Small Interfering RNA Transfections

After seeding into the six-well plates overnight, HCC cells were transiently transfected with 50 nmol control siRNAs and siRNAs targeting *KRT7* or *KRT81* (OBiO Technology, Shanghai) by Lipofectamine 3000 transfection reagent (Invitrogen). 48 h after siRNA transfection, HCC cells were harvested for western blotting and cell migration assays.

### Cell Proliferation Assay

Cell proliferation was measured by Cell Counting Kit-8 (CCK8, Dojindo, Japan) assay according to the manufacturerʼs instructions. Cells were seeded into 96-well plates at a density of 1 × 10^4^ cells/well. Then the cells were incubated with an increasing concentration of SA (25 μΜ, 50 μΜ, 100 μΜ) for 24 and 48 h, respectively. The supernatant was removed and 10 µL CCK-8 solution was added into each well containing 100 µL medium, and further cultured for 2 h at 37°C. The absorbance of each group at 450 nm was detected (*n* = 3) using an absorbance microplate reader (PT-3502C, Potenov, Beijing, China).

### Transwell Migration and Invasion Assays

Transwell plates with 6.5 mm transparent polycarbonate (PC) membrane and matrigel coated transwell plates with 6.5 mm transparent polyester (PET) membrane (3422 and 354480; Corning-Costar, Cambridge, MA, United States) were utilized to detect the migration and invasion ability of cells, respectively. A total of 1 × 10^5^ HepG2 cells and SMMC-7721 cells were resuspended in 100 μL serum-free DMEM and seeded onto the upper chamber at a density of 1 × 10^6^ cells/ml. A total of 600 μL 10%FBS DMEM was added to the lower chamber of transwell chamber. Then cells were cultured for 24 h. The upper surface of the upper chamber was wiped with a cotton swab. The bottom surface of the upper chamber was fixed with methanol and stained with 0.1% crystal violet for 25 min. Cells on the bottom surface of the membrane were imaged and counted in five randomly chosen fields under the microscope (×100) of a DMR inverted microscope (Leica Microsystems, Shanghai, China).

### *In vivo* Orthotopic Xenograft Tumor Model

Male athymic BALB/c nu/nu mice were obtained from the Shanghai Sippr-BK laboratory animal Co. Ltd. Nude mice received care in compliance with the guidelines outlined in the Guide for the Care and Use of Laboratory Animals. HepG2-luciferase cells (2 × 10^6^) were resuspended in 25 μL of PBS/Matrigel (1:1) and inoculated under the capsule of the left hepatic lobe of male BALB/c nude mice at 4 weeks of age. After 2 weeks of tumor cell implantation (Day 0), the mice bearing orthotopic xenografts were randomized into control group (saline, *n* = 6) and SA intervention group (10 mg/kg/d SA, *n* = 6). Daily intraperitoneal injection of SA was given for 14 days. Tumor formation and metastatic progression were monitored and quantified using the Xenogen *In Vivo* Imaging System (IVIS) (Caliper Life Sciences, Hopkinton, MA) 10 min after intraperitoneal injection of 4 mg of luciferin (Gold Biotech). The proportions of intrahepatic metastasis were calculated and compared as previous literature described (Zhao et al., 2019). The procedures were approved by the Animal Care and Use Committee of Shanghai University of Medicine & Health Sciences.

### RNA Extraction, cDNA Library Construction, and Sequencing

HepG2 and SMMC-7721 cells were seeded in 40 mm dishes at a density of 1 × 10^6^ cells/well. Following treatment with 100 μM SA for 24 h, the cells were washed twice with cold PBS and collected by centrifuged for 5 min. Total RNA was extracted using Trizol reagent kit (Invitrogen, Carlsbad, CA, United States) according to the manufacturer’s protocol. RNA quality was assessed on an Agilent 2100 Bioanalyzer (Agilent Technologies, Palo Alto, CA, United States) and checked using RNase free agarose gel electrophoresis. After total RNA was extracted, eukaryotic mRNA was enriched by Oligo (dT) beads, while prokaryotic mRNA was enriched by removing rRNA by Ribo-Zero™ Magnetic Kit (Epicentre, Madison, WI, United States). The enriched mRNA was fragmented into short fragments using fragmentation buffer followed with into cDNA generation by reverse transcriptase with random primers. Then the second strand cDNA were synthesized by DNA polymerase I, RNase H, dNTP and buffer. The cDNA fragments were purified with QiaQuick PCR extraction kit buffer. Then the cDNA fragments were purified with QiaQuick PCR extraction kit (Qiagen, Venlo, The Netherlands), end repaired, poly (A) added, and ligated to Illumina sequencing adapters. The ligation products were size selected by agarose gel electrophoresis, PCR amplified, and sequenced using Illumina HiSeq™ 2500 by Gene Denovo Biotechnology Co. (Guangzhou, China).

### RNA-Seq Data Quality Analysis

In order to ensure data quality for the following analyses, the raw data of 12 samples were firstly filtered through fastp (Chen et al., 2018) (https://github.com/OpenGene/fastp). Finally, clean data were obtained by removing reads containing adapter, reads containing more than 10% of unknown nucleotides (N) and low quality reads containing more than 50% of low quality (Q value ≤20) bases from raw data. At the same time, Q20, Q30, and GC content of the clean data were calculated. All the downstream analyses were based on the clean data with high quality.

### Differentially Expressed Genes Analysis

The differentially expressed genes (DEGs) analysis was performed by DESeq2 (Love et al., 2014) software between two different groups and by edgeR (Robinson et al., 2010) between two samples. We identified genes with a fold change (FC) ≥ 2 and a false discovery rate (FDR) < 0.05 in a comparison as significant DEGs.

### Gene Ontology Enrichment Analysis

Gene Ontology (GO) is an international standardized gene functional classification system which offers a dynamic-updated controlled vocabulary and a strictly defined concept to comprehensively describe properties of genes and their products in any organism (Ashburner et al., 2000). GO has three ontologies: biological process (BP), molecular function (MF), and cellular component (CC). The basic unit of GO is GO term. Each GO term belongs to a type of ontology. The calculated *p* values were gone through FDR Correction, taking FDR ≤0.05 as a threshold. GO terms meeting this condition were defined as significantly enriched GO terms in DEGs. This analysis was able to recognize the main biological functions that DEGs exercise.

### KEGG Pathway Enrichment Analysis

Genes usually interact with each other to play roles in certain biological functions. Pathway based analysis helps to further understand genes biological functions. Kyoto Encyclopedia of Genes and Genomes (KEGG) is the major public pathway related database (Kanehisa and Goto, 2000). Pathway enrichment analysis in DEGs comparing with the whole genome background can identify DEGs into significantly enriched metabolic pathways or signaling pathways. The calculated *p* values were gone through FDR Correction, taking FDR ≤0.05 as a threshold. Pathways meeting this condition were defined as significantly enriched pathways in DEGs.

### Real Time Quantitative RT-PCR

To confirm the transcriptome sequencing data, 9 candidate genes associated with tumor metastasis were selected and validated through RT-qPCR. Total RNA was extracted by using EastepTM Total RNA Super Extraction Kit (promega, Shanghai, China) according to the manufacturer’s instructions and quantified with a Denovix DS-11 Spectrophotometer (Denovix, Inc., Wilmington DE, United States). cDNA was synthesized from total RNA (1 μg: 20 μL final reaction volume) using ReverTra Ace® qPCR RT Master Mix with gDNA Remover (TOYOBO Bio-Technology, CO., Shanghai, China) in a SimpliAmp Thermal Cycler (Applied Biosystems, Thermo Fisher Scientific, Inc., Waltham, MA, United States). A 20 μL PCR reaction system was consist with 2 μL cDNA, 10 μL TB mixture, 0.4 μL forward primer, 0.4 μL reverse primer, 0.4 μL ROX Reference Dye II and 6.8 μL deionized water. After mixing, the PCR reaction was performed using ABI Prism TM 7500 Real-Time qPCR System (Applied Biosystems; Thermo Fisher Scientific, Inc.). The β-actin was used as a house gene to normalize the expression level of the test genes, and the relative gene expression level was analyzed using the 2^−ΔΔCT^ method. All of the samples were analyzed in triplicate. Primers were synthesized by GENEWIZ (Suzhou, China) and were listed in Table 1.

**TABLE 1:** qRT-PCR primer sequence of candidate genes associated with metastasis.

Primer name	Forward primer (5′ to 3′)	Reverse primer (5′ to 3′)
KRT7 (Homo)	ACCAGGAACTCATGAGCGTG	TATTCACGGCTCCCACTCCA
SERPINE2	GATCATCGCCTCCCTGGTTT	AGTCGTTGCTTTGCATGGTT
DKK1	TACCCGGGCGGGAATAAGTA	GGGTACGGCTGGTAGTTGTC
ETV4	GTCTGCGTTGTCCCTGAGAA	CAAGGCCACCAGAAATTGCC
MYEOV	GGCGCCTGTACTGTCTTTCT	ACTGAATTGGTTGGGAGGGC
KRT81	CAGCAGCTGCCGGAAATGTTA	GGGGTCTTTCAAAGTGCAGGA
TNS4	CCCAGTGTCTGATGTCAGCTAT	CTGGAGGAAGAGTTGGCTGG
TNFSF15	GATAAGCCAAGGGCACACCT	GGCCAGGCCTAGTTCATGTT
PTGS2	TGCGCCTTTTCAAGGATGGA	CCCCACAGCAAACCGTAGAT
β-actin	GGGAAATCGTGCGTGACATTAAG	TGTGTTGGCGTACAGGTCTTTG

### Western Blot Analysis

Protein extraction and immunoblot analyses were performed as described below. Cells were lysed in RIPA buffer (EpiZyme, Shanghai, China). The lysates were centrifuged and the supernatants were collected. Cell lysate (20 μg) was separated by SDS–polyacrylamide gel electrophoresis, transferred onto PVDF membranes. After blocking, membranes were incubated with a primary antibody: rabbit monoclonal against SERPINE2, KRT81, KRT7, DKK1, β-Catenin, WNT3A, NF-kB p65, VEGFC, TNFR1, GAPDH (diluted 1:1,000; ABclonal, China), and mouse monoclonal against N-Cadherin, MMP-9 (diluted 1:1,000; Cell Signaling Technology, USA) for overnight at 4°C. After repeated washing, the membranes were incubated with horseradish-peroxidaseconjugated anti-mouse or anti-rabbit secondary antibody (diluted 1:10,000; ABclonal, Shanghai, China). The bands were visualized using the enhanced chemiluminescence (ECL) system (Amersham Pharmacia Biotech). Each experiment was performed in triplicate.

### Statistical Analysis

The results were expressed as the mean ± SEM. The statistical analyses were performed using one-way ANOVA and Student’s t-tests. All statistical analyses were performed using SPSS version 22.0 software (IBM Corp, Armonk, NY, United States) with a statistical significance set at *p* < 0.05.

## Results

### The Inhibitory Effect of Sennoside A (SA) on the Proliferation, Migration and Invasion of HCC *in vitro* and *in vivo*

**FIGURE 1:**
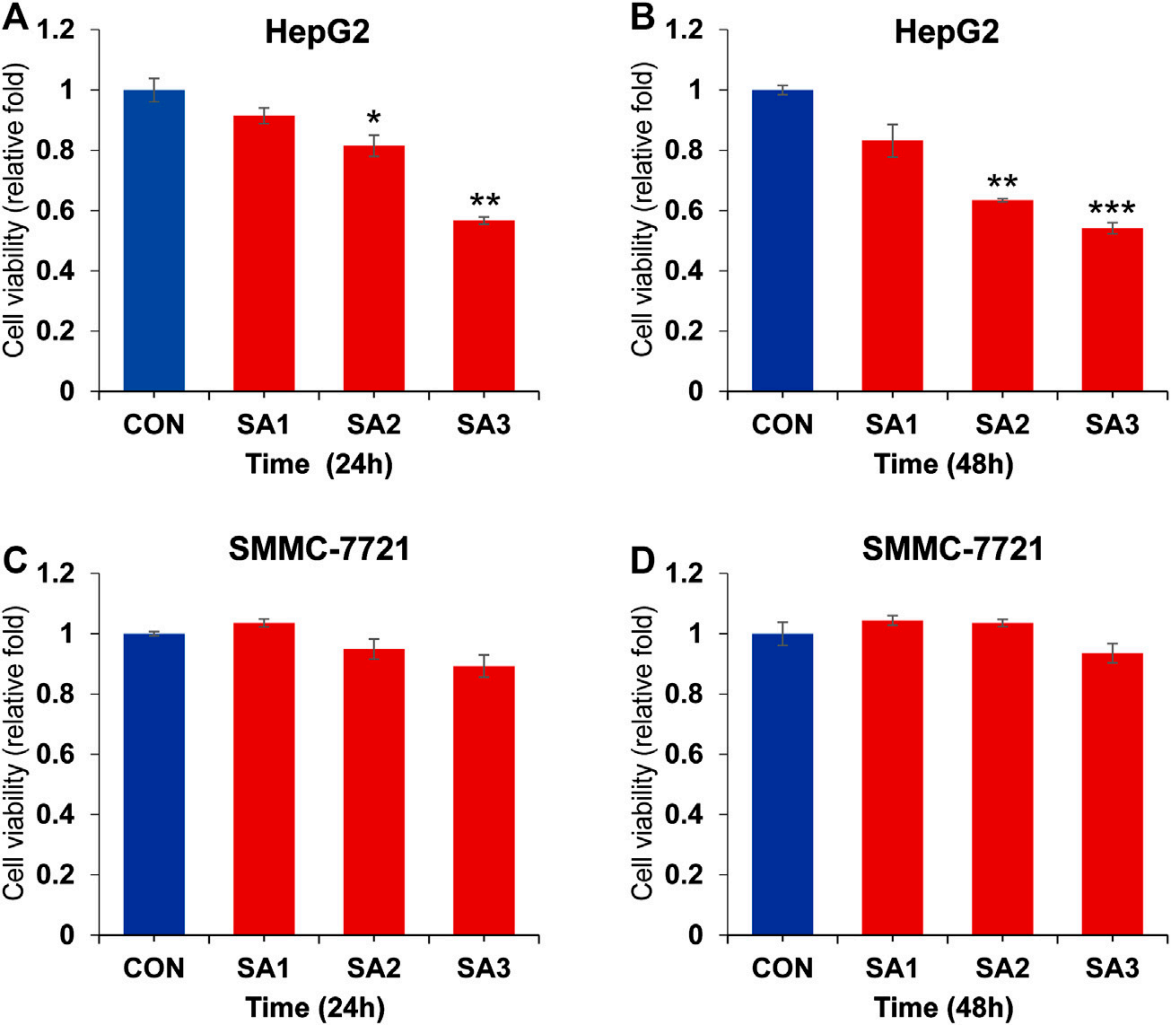
The inhibitory effect of SA on the proliferation of HCC. The cell viability at 24 h **(A)** and 48 h **(B)** in HepG2 cells post SA treatment at indicated dosages. The cell viability at 24 h **(C)** and 48 h **(D)** in SMMC7721 cells post SA treatment at indicated dosages. Data are presented as mean ± SEM (*n* = 3). CON: the control group, SA1: 25 μM SA-treated group, SA2: 50 μM SA-treated group, SA3: 100 μM SA-treated group. *p < 0.05, **p < 0.01, ***p < 0.001 vs. CON.

**FIGURE 2:**
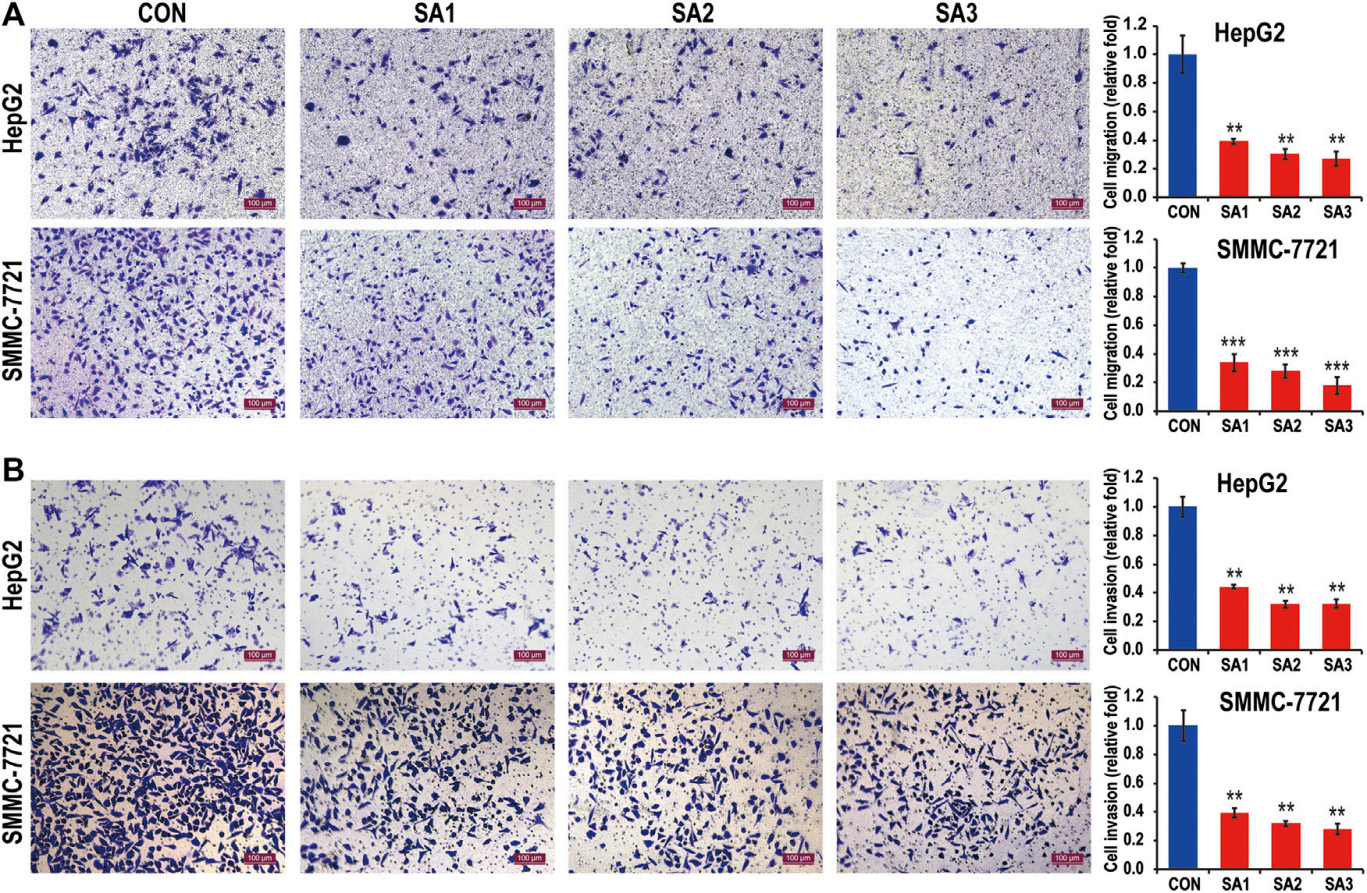
The inhibitory effect of SA on the migration and invasion of HCC. The migration abilities **(A)** and the invasion abilities **(B)** of HepG2 and SMMC7721 cells in response to SA treatment at indicated dosages (original magnification ×100, scale bars 100 μm). Data are presented as mean ± SEM (*n* = 3). CON: the control group, SA1: 25 μM SA-treated group, SA2: 50 μM SA-treated group, SA3: 100 μM SA-treated group. *p < 0.05, **p < 0.01, ***p < 0.001 vs. CON.

To clarify whether the treatment of SA can affect the HCC growth and metastasis, we conducted the CCK8 and Transwell migration and invasion assays to evaluate the potential inhibitory effect of SA in two HCC cell lines, HepG2 and SMMC-7721 cells. As shown in Figure 1, SA treatment had no significant inhibitory effect on SMMC-7721 cell growth, while SA inhibited HepG2 cell growth in a concentration-dependent manner. One possible explanation is that the impact of SA on cell proliferation may vary in different cell types. Meanwhile, compared with control group (CON), SA treatment significantly inhibited migration and invasion in both HCC cell lines and this inhibitory effect was in a dose-dependent manner (Figure 2). To investigate the *in vivo* effect of SA on HCC progression, we established an orthotopic xenograft tumor model by using a HepG2 cell line with stable luciferase expression (HepG2-Luciferase). Tumor formation and metastatic progress were monitored and quantified using the Xenogen *In Vivo* Imaging System (IVIS). The results showed that SA treatment greatly inhibited the growth and intrahepatic metastasis of HepG2-Luciferase cells (Supplementary Figure S1). These results *in vitro* and *in vivo* suggested that SA inhibited cell migration and invasion in HCCs but may suppress HCC cell growth in a cell-type specific manner.

### RNA-Seq Data Generation and Quality Assessment

To identify the target genes downstream of SA-mediated migration and invasion inhibition in HCCs, we performed transcriptome analyses in both cell lines treated with SA. As shown in Tables 2 and 3, the rates of clean reads for all samples exceeded 98%, indicating that the utilization rate of sequencing data was high. The Q20 and Q30 proportion of all samples exceeded 98% and 96% respectively. The rates of reads containing adapter and low quality reads in tested samples were below 0.6%. The rates of reads containing more than 10% of unknown nucleotides (N) and Poly A in all samples were 0%. The data indicated that the quality of the clean reads obtained was high, and the sequence data are highly qualified for the next step in bioinformatics analysis.

**TABLE 2:** Data quality and reference sequence alignment analysis results in HepG2 cell.

Summary	CON-1	CON-2	CON-3	SA-1	SA-2	SA-3
Filter information of reads						
Clean reads (%)	66,794,690 (99.02%)	61,151,782 (99.01%)	64,054,848 (99.01%)	58,283,616 (99.08%)	56,701,380 (99.07%)	56,875,946 (98.95%)
Adapter (%)	319,350 (0.47%)	307,220 (0.5%)	295,212 (0.46%)	233,256 (0.4%)	237,624 (0.42%)	265,254 (0.46%)
Low quality (%)	344,794 (0.51%)	303,164 (0.49%)	345,070 (0.53%)	309,958 (0.53%)	296,674 (0.52%)	340,740 (0.59%)
Poly A (%)	64 (0%)	54 (0%)	57 (0%)	48 (0%)	47 (0%)	73 (0%)
N (%)	0 (0%)	0 (0%)	0 (0%)	0 (0%)	0 (0%)	0 (0%)
Base information after filtration						
Clean base (bp)	9,864,962,546	8,998,168,435	9,444,518,302	8,619,734,484	8,384,852,570	8,388,080,988
Q20 (%)	9,763,073,906 (98.97%)	8,909,709,405 (99.02%)	9,348,071,561 (98.98%)	8,532,861,324 (98.99%)	8,300,240,176 (98.99%)	8,304,449,587 (99.00%)
Q30 (%)	9,485,907,605 (96.16%)	8,665,803,669 (96.31%)	9,085,805,057 (96.20%)	8,296,132,851 (96.25%)	8,069,194,250 (96.24%)	8,084,685,724 (96.38%)
N (%)	34,722 (0.00%)	32,147 (0.00%)	33,181 (0.00%)	30,403 (0.00%)	29,675 (0.00%)	50,784 (0.00%)
GC (%)	5,033,254,008 (51.02%)	4,581,422,632 (50.92%)	4,872,805,882 (51.59%)	4,436,879,561 (51.47%)	4,304,022,675 (51.33%)	4,254,067,037 (50.72%)

**TABLE 3:** Data quality and reference sequence alignment analysis results in SMMC-7721 cell.

Summary	CON-1	CON-2	CON-3	SA-1	SA-2	SA-3
Filter information of reads						
Clean reads (%)	46,516,770 (98.94%)	56,956,274 (99%)	75,635,120 (99%)	56,040,230 (98.86%)	64,223,260 (98.97%)	64,198,644 (99.03%)
Adapter (%)	240,684 (0.51%)	271,368 (0.47%)	371,174 (0.49%)	350,262 (0.62%)	317,546 (0.49%)	270,052 (0.42%)
Low quality (%)	258,088 (0.55%)	304,570 (0.53%)	389,366 (0.51%)	294,458 (0.52%)	352,174 (0.54%)	356,310 (0.55%)
Poly A (%)	50 (0%)	50 (0%)	65 (0%)	56 (0%)	66 (0%)	65 (0%)
N (%)	0 (0%)	0 (0%)	0 (0%)	0 (0%)	0 (0%)	0 (0%)
Base information after filtration						
Clean base (bp)	6,864,610,111	8,412,877,225	11,178,274,636	8,248,324,550	9,480,052,856	9,481,767,152
Q20 (%)	6,798,409,554 (99.04%)	8,329,395,951 (99.01%)	11,068,763,576 (99.02%)	8,165,396,671 (98.99%)	9,384,678,022 (98.99%)	9,382,023,304 (98.95%)
Q30 (%)	6,616,921,614 (96.39%)	8,102,214,678 (96.31%)	10,768,892,966 (96.34%)	7,940,044,070 (96.26%)	9,126,209,503 (96.27%)	9,112,936,040 (96.11%)
N (%)	24,506 (0.00%)	29,628 (0.00%)	40,081 (0.00%)	29,270 (0.00%)	33,642 (0.00%)	33,840 (0.00%)
GC (%)	3,545,531,847 (51.65%)	4,336,106,225 (51.54%)	5,762,331,424 (51.55%)	4,237,625,664 (51.38%)	4,899,660,401 (51.68%)	4,829,798,125 (50.94%)

### The Analysis of Differentially Expressed Genes (DEGs)

**FIGURE 3:**
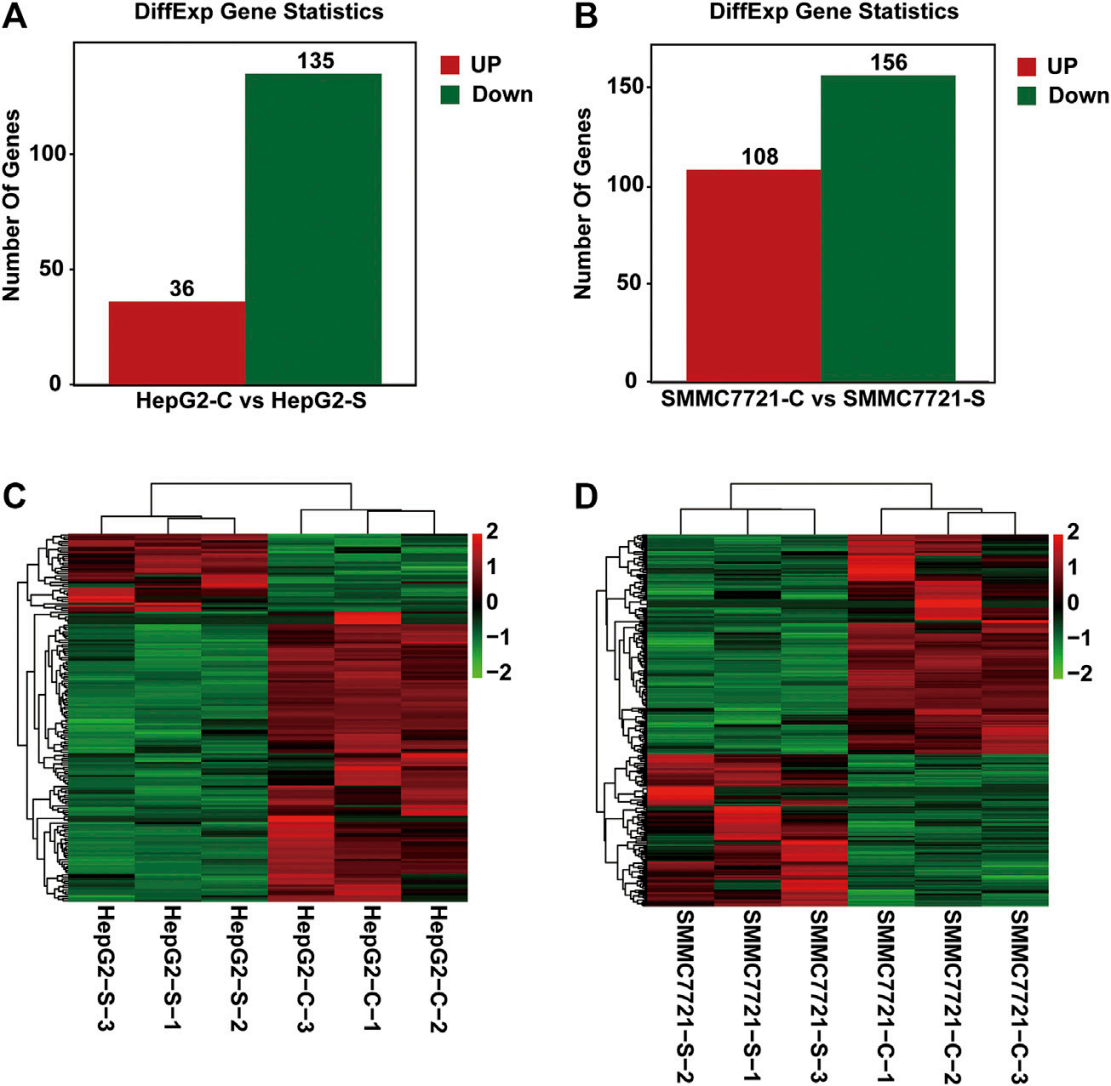
Differentially expressed genes (DEGs) analysis. The distribution pattern of DEGs between the control group (CON) and 100 μM SA-treated group (SA) in HepG2 cells **(A)** and in SMMC7721 cells **(B)**. The heatmap of DEGs between CON and SA groups in HepG2 cells **(C)** and in SMMC7721 cells **(D)**. DEGs were identified with the criteria of FC ≥ 2 and FDR <0.05. Red color or positive number indicates upregulation, and green color or negative number indicates down-regulation.

To identify the differentially expressed genes (DEGs), the fragments per kilobase of exon per million fragments mapped (FPKM) method was utilized to calculate the gene expression levels between the control and SA-treated samples. The gene differential expression in all samples was comprehensively analyzed with the criteria of FC ≥ 2 and FDR <0.05. Total 171 and 264 DEGs were respectively identified in HepG2 and SMMC-7721 cells (Figures 3A,B). Compared with the control group, 36 upregulated and 135 down-regulated genes were identified in HepG2 cells treated with SA (Figure 3A). In SMMC-7721 cells, there were 108 upregulated and 156 down-regulated genes in the SA treatment group compared with the control group (Figure 3B). Hierarchical clustering analysis of those DEGs was performed to group similar samples and genes in HepG2 and SMMC-7721 cells. These results were graphically depicted using heat map and dendrogram (Figures 3C,D). DEGs identified in biological replicates were clustered together, indicating good reproducibility of the SA treatment.

### Analysis of DEGs With Common Expression Changes in Response to SA Treatment

**FIGURE 4:**
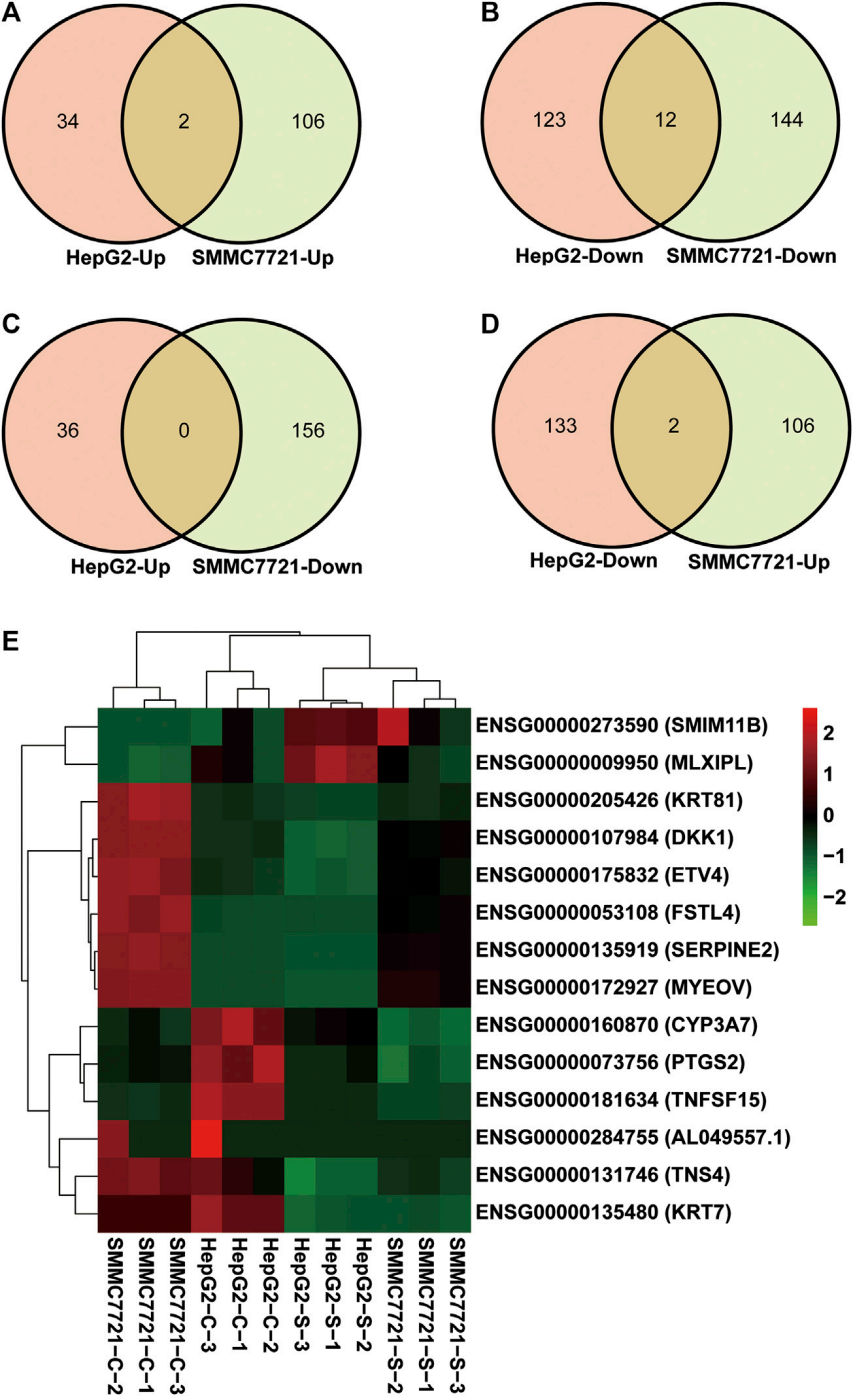
Identification of DEGs with common expression changes in response to SA treatment. Venn diagram of upregulated common DEGs **(A)** and downregulated common DEGs **(B)** between HepG2 and SMMC7721 cells. Venn diagram of common DEGs showing opposite expression changes between HepG2 and SMMC7721 cells **(C**, **D)**. The heatmap representing the expression levels of total 14 common DEGs between HepG2 and SMMC7721 cells **(E)**. Red color or positive number indicates upregulation, and green color or negative number indicates down-regulation.

To find the target genes of SA in inhibiting migration and invasion of HCCs, we selected DEGs with common expression changes in both cell lines upon SA treatment (Figure 4 and Table 4). Venn diagram analysis of the DEGs indicated that 2 DEGs and 12 DEGs were respectively up-regulated or down-regulated in both HepG2 and SMMC-7721 cells (Figures 4A,B). The 2 upregulated genes were ENSG00000273590 (*SMIM11B*) and ENSG00000009950 (*MLXIPL*), and the 12 down-regulated genes were ENSG00000160870 (*CYP3A7*), ENSG00000107984 (*DKK1*), ENSG00000073756 (*PTGS2*), ENSG00000131746 (*TNS4*), ENSG00000175832 (*ETV4*), ENSG00000135919 (*SERPINE2*), ENSG00000135480 (*KRT7*), ENSG00000053108 (*FSTL4*), ENSG00000172927 (*MYEOV*), ENSG00000205426 (*KRT81*), ENSG00000181634 (*TNFSF15*) and ENSG00000284755 (*AL049557.1*). There were no DEGs which were up-regulated in HepG2 cells showing down-regulation in SMMC-7721 cells (Figure 4C). However, 2 DEGs (*XLOC_016469* and *XLOC_014092*) down-regulated in HepG2 cells without annotation showed up-regulation in SMMC-7721 cells (Figure 4D). Hierarchical clustering analysis of those 14 common DEGs indicated good reproducibility of the SA treatment in both cell lines (Figure 4E).

**TABLE 4:** DEGs with a consistent trend in both HepG2 and SMMC-7721 cells.

Gene ID	Description	Symbol	HepG2		SMMC7721		Variation trend
			Pvalue	FDR	Pvalue	FDR	
ENSG00000273590	Small integral membrane protein 11B	SMIM11B	0.000239975	0.007964029	3.81E−08	1.68E−06	Up
ENSG00000009950	MLX interacting protein like	MLXIPL	3.47E-07	2.86E−05	6.59E−10	4.64E−08	Up
ENSG00000160870	Cytochrome P450 family 3 subfamily A member 7	CYP3A7	1.23E-05	0.00070067	4.65E−05	0.000801757	Down
ENSG00000107984	Dickkopf WNT signaling pathway inhibitor 1	DKK1	3.55E−21	1.97E−18	9.33E−52	1.68E−48	Down
ENSG00000073756	Prostaglandin-endoperoxide synthase 2	PTGS2	3.64E−12	7.15E−10	5.02E−06	0.000123299	Down
ENSG00000131746	Tensin 4	TNS4	2.96E−13	6.87E−11	2.11E−24	7.62E−22	Down
ENSG00000175832	ETS variant 4	ETV4	5.28E−19	2.45E−16	2.22E−62	5.72E−59	Down
ENSG00000135919	Serpin family E member 2	SERPINE2	3.41E−29	4.38E−26	3.72E−115	3.35E−111	Down
ENSG00000135480	Keratin 7	KRT7	1.63E−58	9.05E−55	9.47E−133	1.71E−128	Down
ENSG00000053108	Follistatin like 4	FSTL4	8.87E−08	8.17E−06	9.85E−90	4.44E−86	Down
ENSG00000172927	—	MYEOV	1.86E−16	6.10E−14	3.73E−43	4.80E−40	Down
ENSG00000205426	Keratin 81	KRT81	3.19E−11	5.55E−09	3.86E−82	1.39E−78	Down
ENSG00000181634	TNF superfamily member 15	TNFSF15	9.88E−89	1.65E−84	2.70E−09	1.62E−07	Down
ENSG00000284755	Inka box actin regulator 2	AL049557.1	0.000275314	0.008793691	0.001018577	0.009862299	Down

### GO Enrichment Analysis of DEGs

**FIGURE 5:**
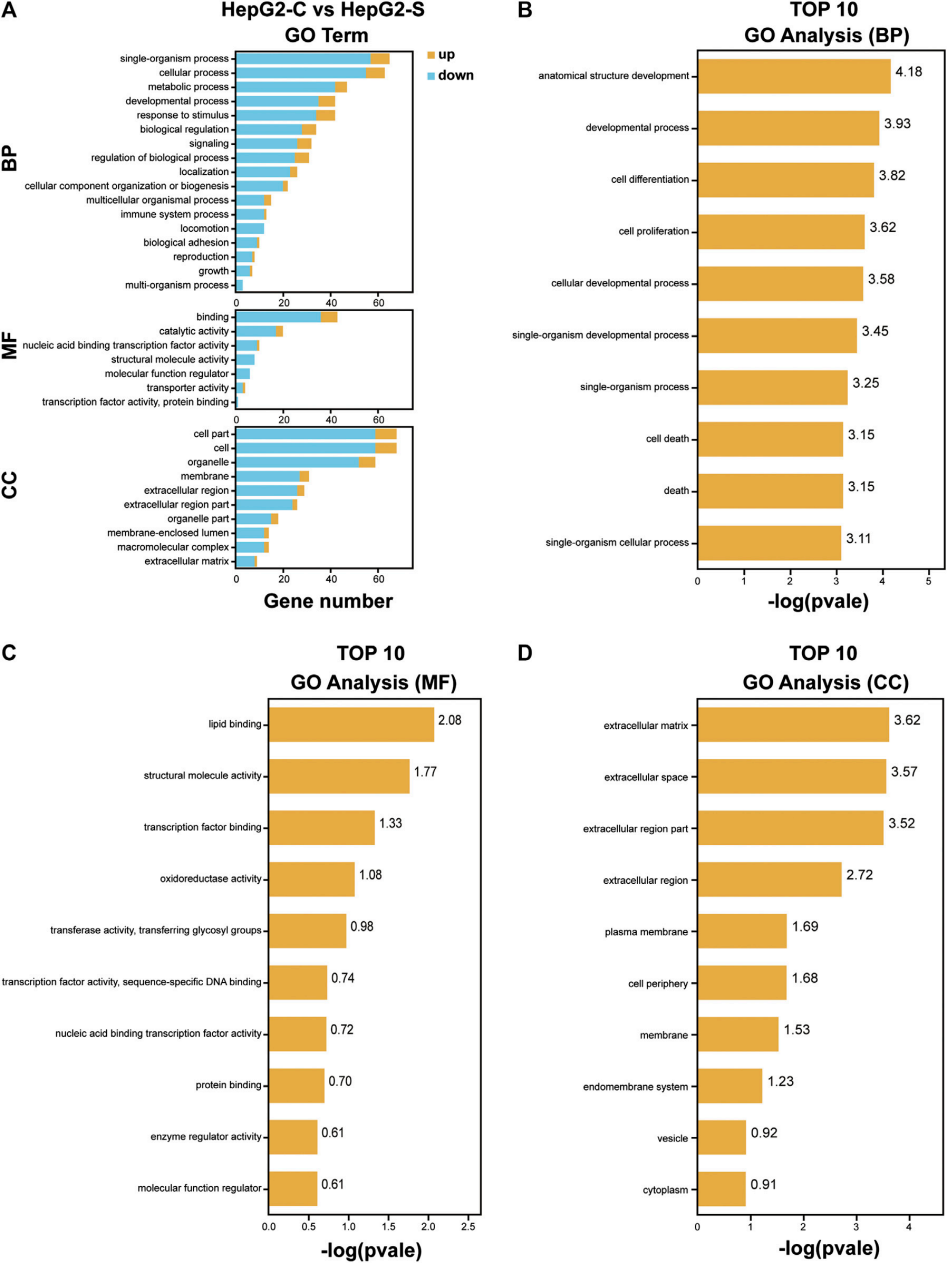
GO enrichment analysis of DEGs in HepG2 cells. The annotation and classification of GO functional enrichment analysis with the DEGs in HepG2 cells **(A)**. The top 10 functional enriched classes of DEGs in HepG2 cells annotated by biological processes (BP) **(B)**, molecular function (MF) **(C)**, and cell components **(D)** sub-ontologies of GO enrichment analysis.

**FIGURE 6:**
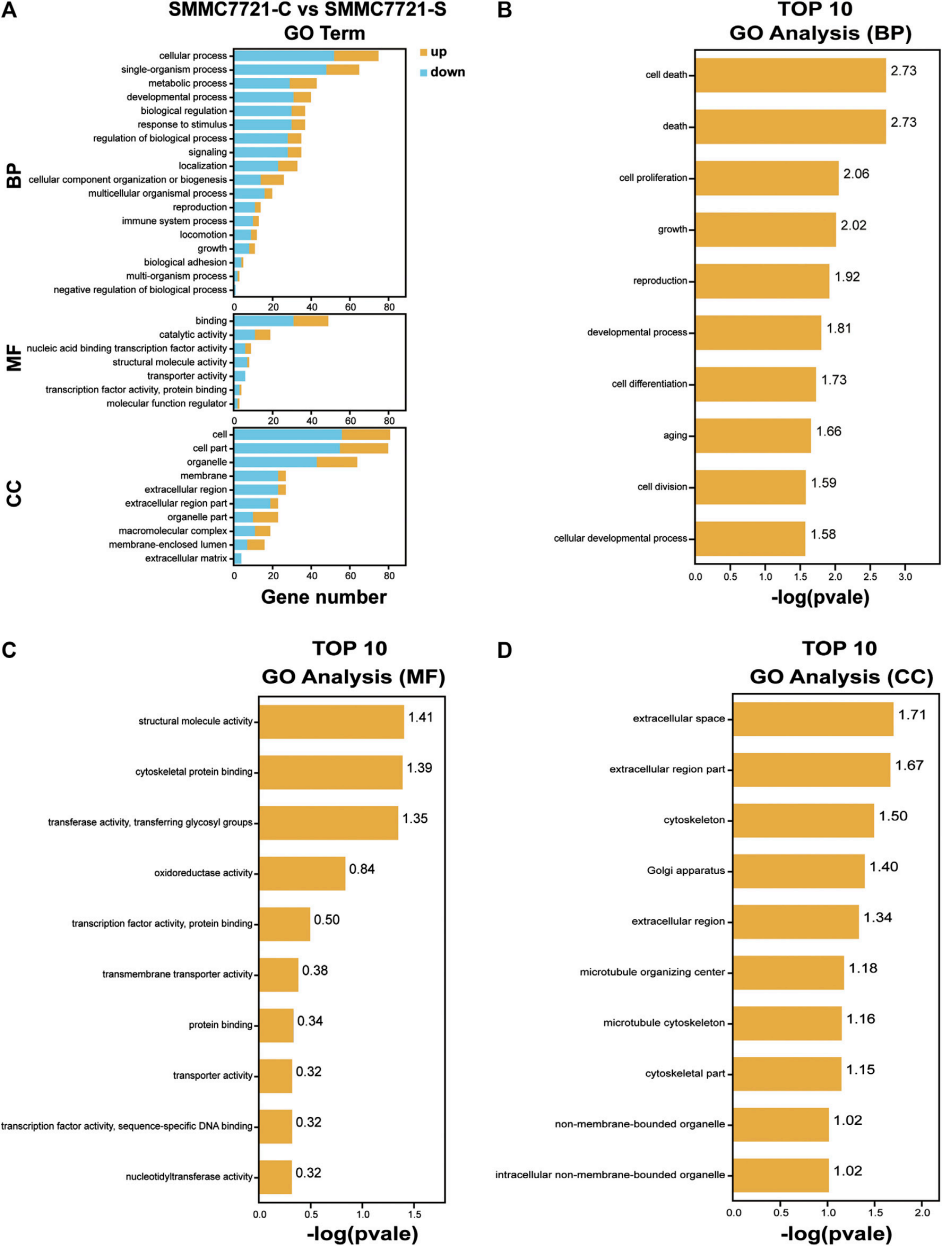
GO enrichment analysis of DEGs in SMMC7721 cells. The annotation and classification of GO functional enrichment analysis with the DEGs in SMMC7721 cells **(A)**. The top 10 functional enriched classes of DEGs in SMMC7721 cells annotated by biological processes (BP) **(B)**, molecular function (MF) **(C)**, and cell components (CC) **(D)** sub-ontologies of GO enrichment analysis.

To investigate the function of the identified DEGs, we conducted GO enrichment analyses on the 171 DEGs in HepG2 cells and the 264 DEGs in SMMC-7721 cells (Figures 5A,6A). The results indicated that the 171 DEGs in HepG2 cells were mainly enriched in the biological processes (BP) associated with anatomical structure development, developmental process, cell differentiation and cell proliferation, whereas the 264 DEGs in SMMC-7721 cells were mainly enriched in the biological processes (BP) associated with cell death, death, cell proliferation, and growth (Figures 5B,6B). As for molecular function (MF), the DEGs in HepG2 were mainly enriched in lipid binding, structural molecule activity, transcription factor binding and oxidoreductase activity (Figure 5C), and the DEGs in SMMC-7721 cells were mainly enriched in structural molecule activity, cytoskeletal protein binding, transferase activity and oxidoreductase activity (Figure 6C). Additionally, the results for the cellular component (CC) analysis showed that the DEGs in HepG2 were mainly enriched in extracellular matrix, extracellular space, extracellular region part and extracellular region (Figure 5D), whereas the DEGs in SMMC-7721 cells were mainly enriched in extracellular space, extracellular region part, cytoskeleton and Golgi apparatus (Figure 6D).

### KEGG Pathway Enrichment Analysis of DEGs

**FIGURE 7:**
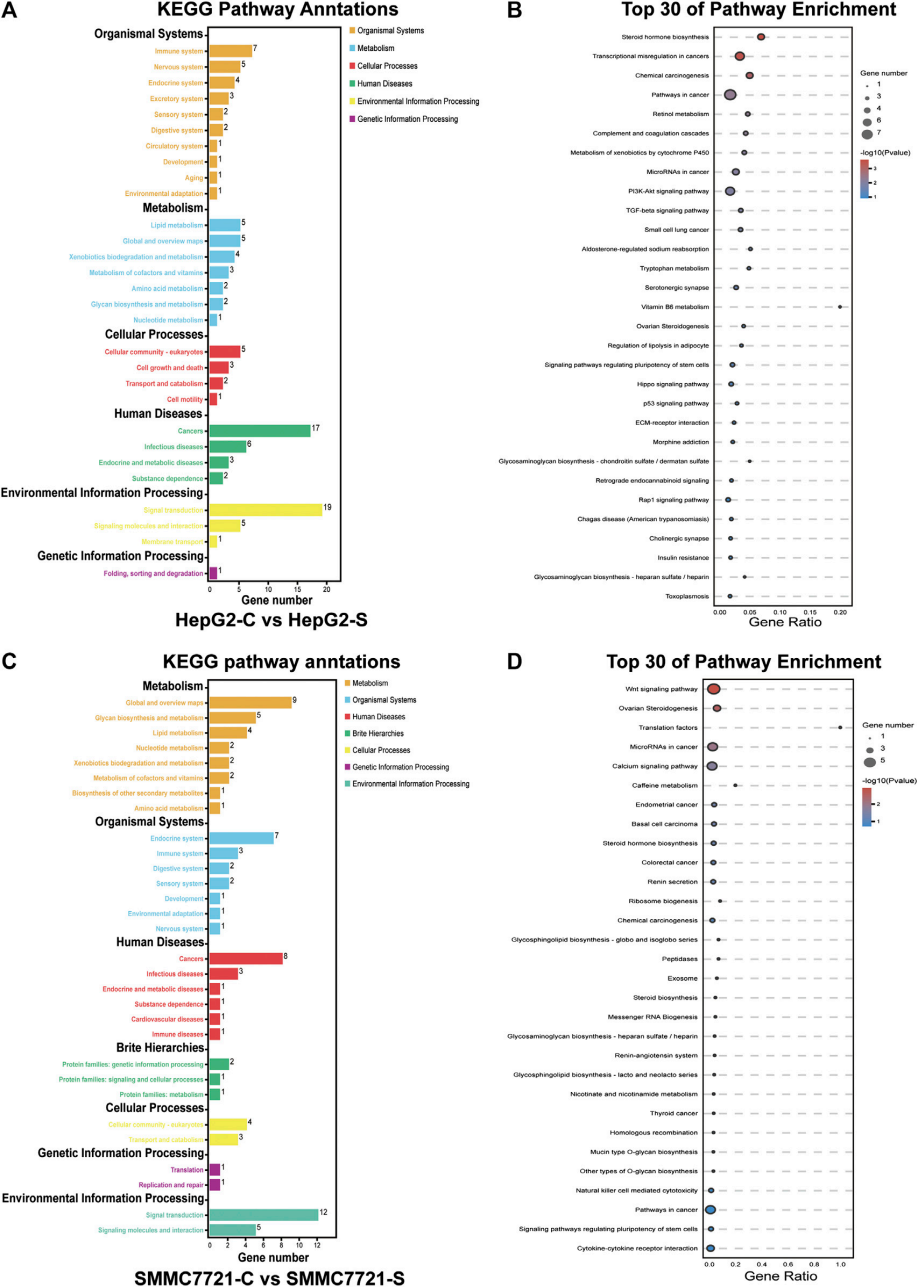
KEGG pathway enrichment analysis of DEGs. KEGG pathway annotation and classification of DEGs in HepG2 cells **(A)**. Scatterplot of the top 30 KEGG enrichment pathways of DEGs in HepG2 cells **(B)**. KEGG pathway annotation and classification of DEGs in SMMC7721 cells **(C)**. Scatterplot of the top 30 KEGG enrichment pathways of DEGs in SMMC7721 cells **(D)**.

To investigate which pathways are involved in SA mediated inhibition on migration and invasion in HCCs, we performed Kyoto Encyclopedia of Genes and Genomes (KEGG) pathway analysis by mapping the DEGs to the KEGG pathway. The DEGs in HepG2 cells were clustered into six main categories, including organismal systems, metabolism, cellular process, human diseases, environmental information processing, and genetic information processing, which were further divided into 29 subcategories (Figure 7A). The DEGs in SMMC-7721 cells were clustered into seven main categories including organismal systems, metabolism, human diseases, environmental information processing, cellular process, and brite hierarchies, which were further divided into 30 subcategories (Figure 7C). The enriched pathways were then analyzed with a significance test, and the top 30 pathways were found to be significantly enriched by pairwise comparisons in HepG2 and SMMC-7721 cells (*p* ≤ 0.05) (Figures 7B,D). The main enriched pathways in HepG2 cells were represented including pathways related to transcriptional misregulation in cancers, pathways in cancers, microRNAs in cancers, PI3K-Akt signaling pathway, TGF-β signaling pathway, Hippo signaling pathway, p53 signaling pathway, and Rap1 signaling pathway (Figure 7B). In SMMC-7721 cells, the main enriched pathways included pathways in cancers, microRNAs in cancers, Wnt signaling pathway and pathways in cancers (Figure 7D). Among these enriched pathways, the pathways of PI3K-Akt signaling, TGF-β signaling, Hippo signaling, p53 signaling, Rap1 signaling and Wnt signaling have been reported to be associated to HCC metastasis.

### GO and KEGG Enrichment Analyses of DEGs With Common Expression Changes

**FIGURE 8:**
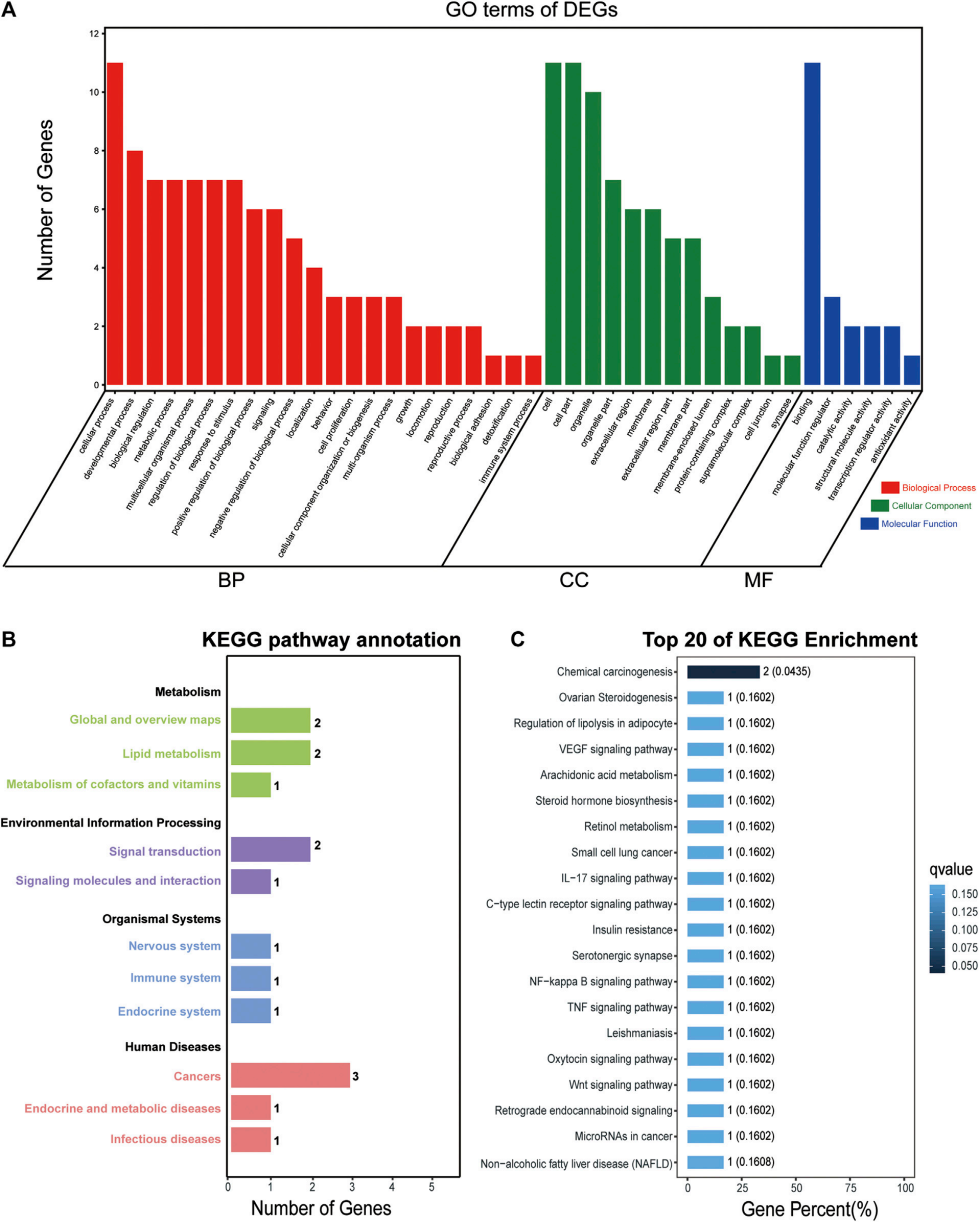
GO and KEGG enrichment analyses of DEGs with common expression changes in both cell lines. GO analysis of the 14 DEGs with common expression changes in both cell lines **(A)**. Genes were classified into biological processes (BP), cell components (CC), and molecular function (MF). The right *y* axis indicates the number of genes in a category. KEGG pathway annotation and classification **(B)** and the top 20 KEGG pathway analysis **(C)** of the 14 common DEGs in both cell lines.

To reveal the biological function and signaling pathways of the molecular targets of SA in inhibiting migration and invasion of HCC, we performed GO and KEGG pathway analyses of the 14 DEGs with common expression changes in HepG2 and SMMC-7721 cells in response to SA treatment. As shown in Figure 8A, the cellular process, developmental process and biological regulation were the main representative functions in BP; the cell, cell parts and organelles were the most significant terms in CC; the binding, molecular function regulator, and catalytic activity were the subcategory of highest percentages in MF. KEGG pathway enrichment analyses clustered all the 14 common DEGs into four major categories, metabolism, environmental information processing, organismal systems, and human diseases, which could be further divided into 11 subcategories (Figure 8B). The top 20 of pathway enrichment were determined by KEGG analysis (Figure 8C). The KEGG pathway analysis showed that the pathways of the 14 common DEGs were mainly enriched in the VEGF signaling pathway, TNF signaling pathway, NF-κB signaling pathway and Wnt signaling pathway, which were all associated with the migration and invasion of HCC.

### Validation of Metastasis-Related DEGs and KEGG Enrichment Pathways

**FIGURE 9:**
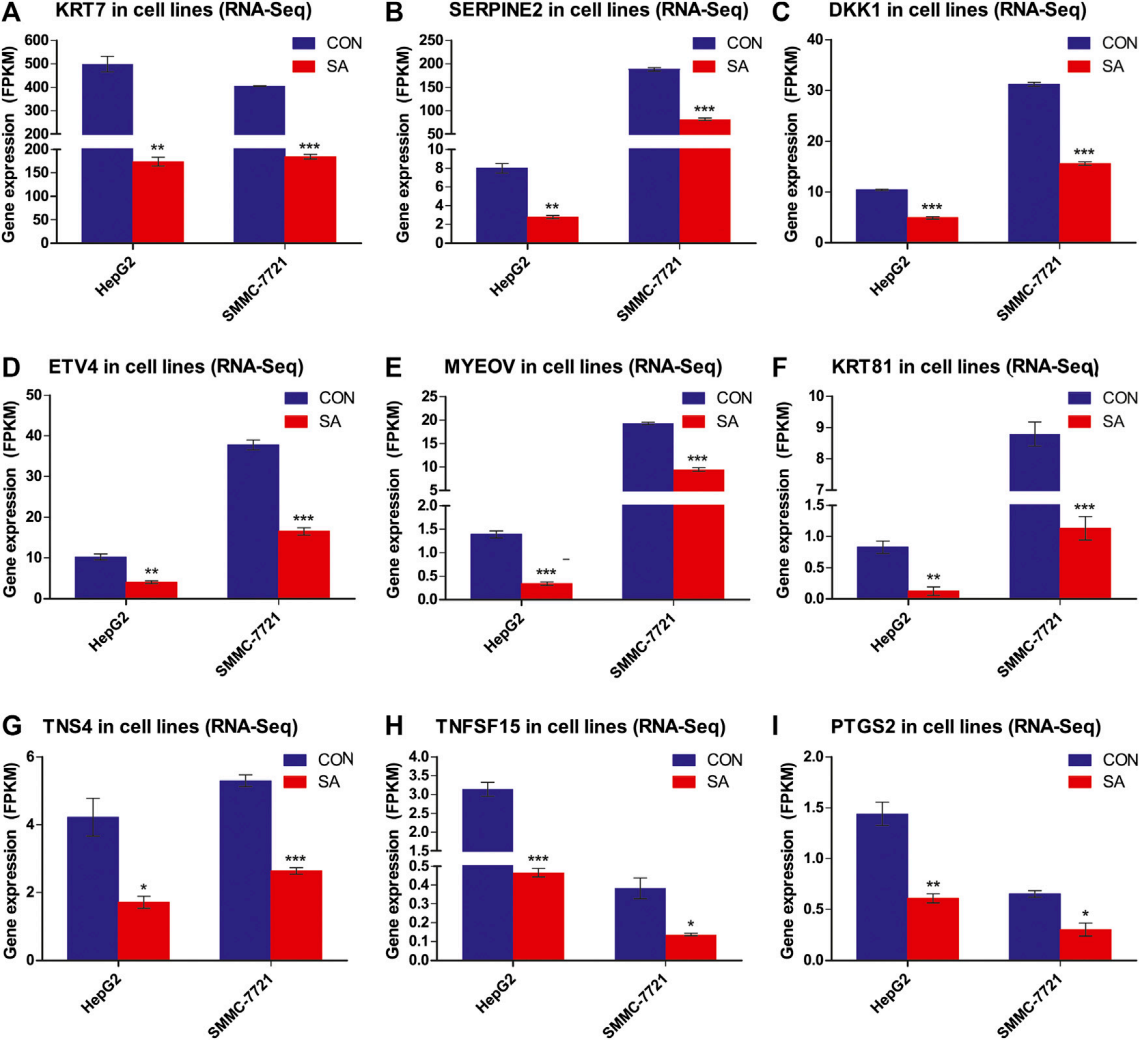
Differential Expression of the 9 metastasis-related DEGs. Gene expression levels of the 9 metastasis-related DEGs in RNA-Seq data (FPKM method) (A-I). Data are presented as mean ± SEM (n = 3). CON: the control group, SA: 100 μM SA-treated group. *p < 0.05, **p < 0.01, ***p < 0.001 vs. CON.

**FIGURE 10:**
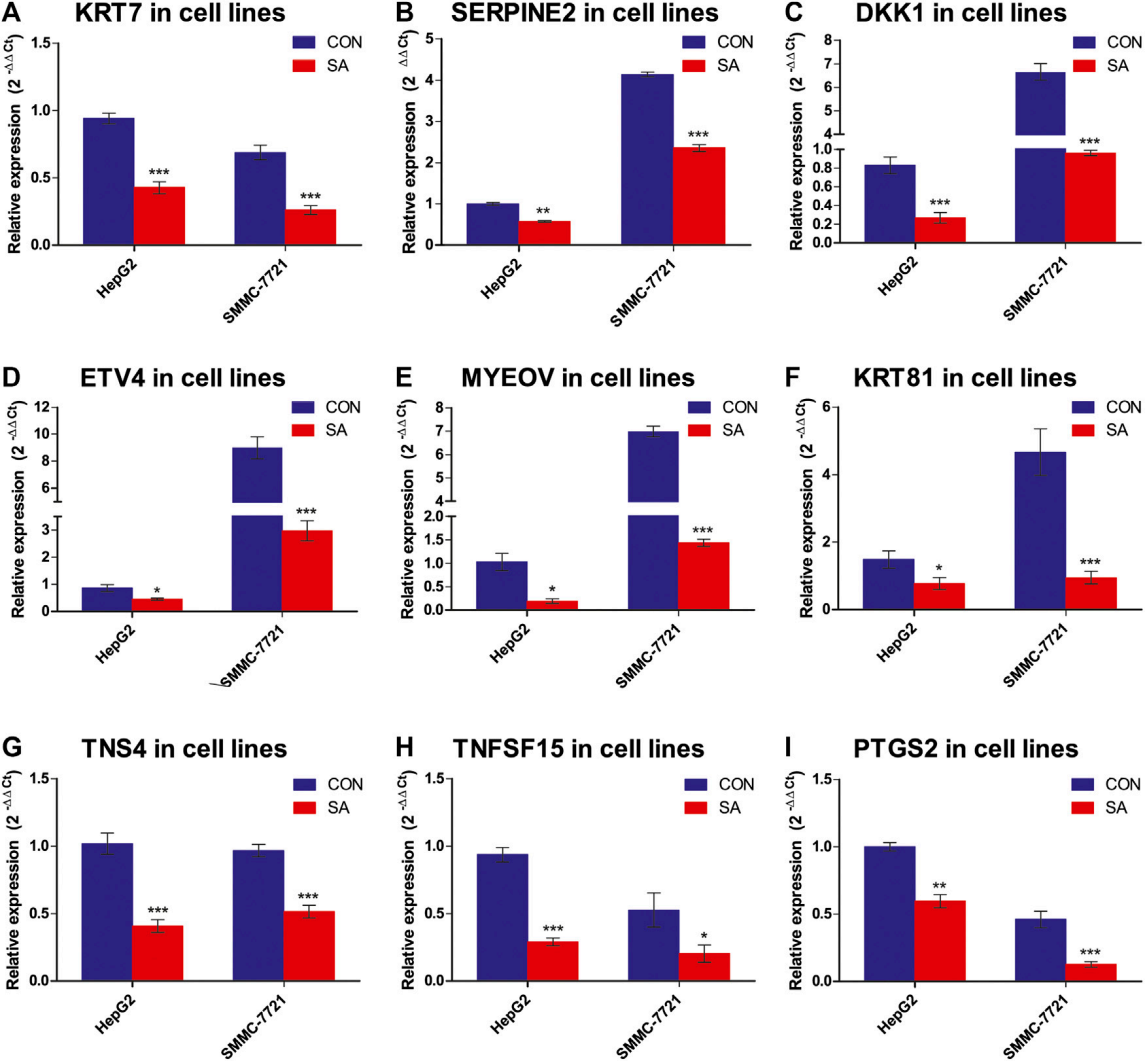
Expression validation of the 9 metastasis-related DEGs via RT-qPCR. The mRNA levels of the 9 metastasis-related DEGs were validated by RT-qPCR (2−ΔΔCT method) (A-I). The housekeeping gene β-actin was used to normalize the relative expression level. Data are presented as mean ± SEM (*n* = 3). CON: the control group, SA: 100 μMSA-treated group. *p < 0.05, **p < 0.01, ***p < 0.001 vs. CON.

Among the 14 common DEGs, 9 DEGs are associated with tumor metastasis. They are *KRT7*, *SERPINE2*, *DKK1*, *ETV4*, *MYEOV*, *KRT81*, *TNS4*, *TNFSF15* and *PTGS2*. The RNA-seq data showed that the 9 metastasis-associated DEGs were down-regulated in both cell lines upon SA treatment (Figure 9). Our RT-qPCR assays also validated their downregulation in both cell lines in response to SA treatment (Figure 10). Meanwhile, western blot analysis confirmed that SA downregurated metastasis-related DEGs, such as *DKK1*, *KRT7*, *KRT81* and *SERPINE2* (Supplementary Figure S2A–C), and inhibited KEGG enrichment metastasis-related pathways (Wnt signaling pathway, TNF signaling pathway, VEGF signaling pathway, and NF-κB signaling pathway) (Supplementary Figure S2D–F). To further investigate which genes contribute to SA-mediated inhibition on HCC cell migration, we examined the regulatory effects of *KRT7* and *KRT81* by knocking down *KRT7* and *KRT81* in HepG2 and SMMC-7721 cells. Our results suggested that the downregulation of *KRT7* and *KRT81* could inhibit HCC metastasis (Supplementary Figure S3).

## Discussion

Metastasis is a highly inefficient multistep process, including invasion of local stroma, intravasation into the bloodstream and/or lymphatic system, and extravasation into a secondary tissue, extravasation to colonize and thrive at distant sites (Strilic and Offermanns, 2017; Gao et al., 2019), and is the leading cause of cancer-associated deaths. This study provided evidence for the first time showing that SA was able to inhibit the migration and invasion in HepG2 and SMMC-7721 cells. To find the potential molecular targets of SA for its inhibitory effects on HCC metastasis, we conducted transcriptome analyses by RNA-Seq in HepG2 and SMMC-7721 cells treated with SA at 100 μΜ. By comparison of control and SA treatment groups, we identified 171 and 264 DEGs in HepG2 and SMMC-7721 cells respectively based on the criteria of FC ≥ 2 and FDR ≤0.05. Among these DEGs, *SMIM11B* and *MLXIPL* were up-regulated whereas *CYP3A7*, *DKK1*, *PTGS2*, *TNS4*, *ETV4*, *SERPINE2*, *KRT7*, *FSTL4*, *MYEOV*, *KRT81*, *TNFSF15* and *AL049557.1* were downregulated in HepG2 and SMMC-7721 cells upon SA treatment. Notably, 9 overlapping down-regulated DEGs, *KRT7*, *SERPINE2*, *DKK1*, *ETV4*, *MYEOV*, *KRT81*, *TNS4*, *TNFSF15* and *PTGS2*, have been reported to be involved in metastasis in many types of cancers. Additionally, the expression changes of these 9 metastasis-related DEGs were verified via RT-qPCR analysis. Both the RNA-seq results and RT-qPCR results showed that the expression of 9 metastasis-related DEGs was significantly down-regulated in the SA-treated groups compared with the CON groups. Finnaly, we found that SA downregulated protein levels of metastasis-related DEGs, including *KRT7*, *SERPINE2*, *DKK1*, *KRT81*, and confirmed that the downregulation of *KRT7* and *KRT81* could inhibit HCC metastasis by employing siRNA-mediated downregulation of *KRT7* and *KRT81*.

Among these 9 metastasis-related DEGs, keratin 7 (*KRT7*) was the most highly expressed gene in HepG2 and SMMC-7721 cells. *KRT7* belongs to type II cytokeratin, which is the component of cytoskeleton and epithelial intermediate filaments, and acts as a membrane-cytoskeletal linker which contributes to regulation of cell adhesion (Helfand et al., 2004; Sano et al., 2010). Previous study indicated that *KRT7* showed enhanced expression levels in several cancer types, such as colorectal carcinoma, esophageal squamous cell carcinoma and gastric cancer, and associates with metastasis (Sano et al., 2010; Huang et al., 2016; Chen et al., 2020). Besides, keratin 81 (*KRT81*), a type II hair keratin, has been reported to be over-expressed in the breast cancer cells and contributed to their invasiveness (Nanashima et al., 2017). Serpin family E member 2 (*SERPINE2*), a member of the serine protease inhibitor superfamily, is a secreted protein with anti-serine protease activity against thrombin, urokinase, plasminogen and other serine proteinases. SERPINE2 played an important role in metastasis in different tumors including breast cancer, melanoma, and esophageal squamous cell carcinoma (Smirnova et al., 2016; Wu, 2016; Zhang et al., 2020). Numerous studies have shown that Dickkopf WNT signaling pathway inhibitor 1 (*DKK1*), a secreted inhibitor of canonical Wnt signaling and osteoblast differentiation, was implicated in the migration and invasion of HCC, cholangiocarcinoma and breast cancer (Chen et al., 2013; Shi et al., 2013; Zhuang et al., 2017). ETS variant transcription factor 4 (*ETV4*) was also reported to be highly expressed in breast cancer and prostate cancer, which was associated with distant metastasis of prostate cancer through PI3-kinase and Ras signaling (de Launoit et al., 2000; Aytes et al., 2013). Myeloma overexpressed (*MYEOV*) acted as an amplified competing endogenous RNA in promoting metastasis by activating TGF-β pathway in non-small cell lung cancer and served as the potential therapeutic target (Fang et al., 2019).

In addition to above relatively high expression DEGs, another 3 less expressed DEGs have also been reported to associated with cancer metastasis. Tensin 4 (*TNS4*) induced epithelial-mesenchymal transition and metastasis by activating the expression of TGF-β1 in lung adenocarcinoma cancer cells (Lu et al., 2018). Tumor necrosis factor receptor superfamily member 25 (*TNFRSF25*) promoted lymphatic metastasis via VEGF signaling pathway in a mouse model of lung cancer (Qin et al., 2018). Prostaglandin-endoperoxide synthase 2 (*PTGS2*) might mediate the CXCR2 signaling to inversely control the breast cancer metastasis and chemoresistance through the regulation of EMT, apoptosis, and senescence (Xu et al., 2018).

Pathway enrichment analysis is considered to simplify data interpretation and to provide vigorous results, depending on the existing databases. In our study, several main pathways were found to be involved in the inhibitory effect of SA on the metastasis of HCC and associated with identified metastasis-related DEGs, including Wnt signaling pathway, TNF signaling pathway, VEGF signaling pathway, and NF-κB signaling pathway. Then, we confirmed that SA inhibited the activation of KEGG enrichment metastasis-related pathways (Wnt, TNF, VEGF, and NF-κB signaling pathways). Wnt signaling pathway is one of the key cascades regulating cancer progression, and has been reported to play an important role in metastasis of many tumors including HCC, non-small cell lung cancer and colorectal cancer (Lin et al., 2017; Yang et al., 2017; Zhang et al., 2018). Recent observations suggest that VEGF signaling pathway might promote tumor metastasis in many tumors including gastric cancer, HCC and breast cancer (Zhang et al., 2017; Chen et al., 2019; Wang et al., 2019). Nuclear factor-kB (NF-κB) activated signaling pathway have been linked with proliferation, angiogenesis, invasion and metastasis in many tumors including breast cancer, prostate cancer and HCC (Sung et al., 2008; Liu et al., 2015; Ren et al., 2017; Wang et al., 2018). TNF, tumor necrosis factor, which is involved in many diseases including cancer, diabetes, and inflammatory bowel diseases. TNF Ligand binding to TNFR1 and TNFR2 leads to the activation of NF-κB, which was associated with modulating inflammatory mediators and growth factors, cell survival proliferation and migration, the regulatory T-cell function (Chen and Goeddel, 2002; Oshima et al., 2014; Lebrec et al., 2015).

In summary, we firstly demonstrated that SA can inhibit the migration and invasion in HCC cells. RNA-seq data analysis identified 9 potential HCC metastasis-related DEGs which may be regulated by SA in HCCs. Moreover, we found that SA downregulated metastasis-related DEGs and inhibited the activation of KEGG enrichment metastasis-related pathways (Wnt, TNF, VEGF, and NF-κB signaling pathways). Finnaly, we confirmed that the downregulation of *KRT7* and *KRT81* could inhibit HCC metastasis. Although further studies are necessary to clarify its specific mechanisms of overall anticancer effect in HCC, our results provided novel evidence on the understanding of the potential targets of SA to inhibit HCC metastasis.

## Data Availability

The datasets generated for this study can be found in NCBI BioProject (PRJNA643657).
