# Supplementary material for: Transcriptome Analysis of the Inhibitory Effect of Sennoside A on the Metastasis of Hepatocellular Carcinoma Cells
Source: Front Pharmacol. 2021 Jan 12;11:566099. doi: 10.3389/fphar.2020.566099 (PMC7942274; doi:10.3389/fphar.2020.566099)
Supplement: Supplementary file 1 [file datasheet1.pdf]

Supplementary

Figure S1. The luciferase signals of the orthotopic xenograft tumor mice.
(A) Representative images of luciferase expression obtained immediately before the treatment (Day 0) and after receiving treatment of SA for 2 weeks (Day 14); (B) The proportions of intrahepatic metastasis. The colored region represents the fluorescence signal of HepG2 cells in nude mice and the proportions of intrahepatic metastasis were calculated and compared. CON: the control group (saline), SA: 10 mg/kg/d SA intervention group.

Figure S2. The regulatory effects of SA on metastasis-related DEGs and KEGG enrichment metastasis-related pathways.
(A) Protein expression levels of EMT markers (MMP-9, N-cadherin) and metastasis-related DEGs (DKK1, KRT7, KRT81 and SERPINE2) in HCC cells. (B) and (C) The densitometry analysis of protein expression in (A). (D) Protein expression levels of markers of metastasis-related pathways (Wnt signaling pathway, TNF signaling pathway, VEGF signaling pathway, and NF-κB signaling pathway) in HCC cells. (E) and (F) The densitometry analysis of protein expression in (D). Data are presented as mean ± SEM (n=3). CON: the control group, SA: 100μM SA-treated group. $^{*}P < 0.05$, $^{**}P < 0.01$, $^{***}P < 0.001$ vs. CON.

Figure S3. The effects of KRT7 and KRT81 on HCC metastasis.
(A) The migration abilities of HCC cells after KRT7 and KRT81 knockdown (original magnification ×100, scale bars 100μm). (B) The quantitative analysis of cell migration in (A). (C) Protein expression levels of KRT7 and EMT markers MMP-9 in HCC cells after KRT7 knockdown. (D) Protein expression levels of KRT81 and EMT markers MMP-9 in HCC cells after KRT81 knockdown. Data are presented as mean ± SEM (n=3). si-NC: negative control group, si-KRT7: siRNA targeting KRT7 group, si-KRT81: siRNA targeting KRT81 group. $^{*}P < 0.05$, $^{**}P < 0.01$, $^{***}P < 0.001$ vs. CON.
