# Supplementary figures and images for: Transcriptome Analysis of the Inhibitory Effect of Sennoside A on the Metastasis of Hepatocellular Carcinoma Cells
Source: Front Pharmacol. 2021 Jan 12;11:566099. doi: 10.3389/fphar.2020.566099 (PMC7942274; doi:10.3389/fphar.2020.566099)

**A****CON****SA****Day 0**

ROI 1=1.419e+07

ROI 2=9.444e+06

PKI20181212115239\_001A

PKI20181212112409\_001A

**Day 14**

ROI 3=5.287e+07

ROI 4=4.892e+04

PKI20181226200603\_001A

PKI20181226200733\_001A

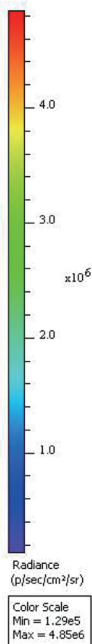**B**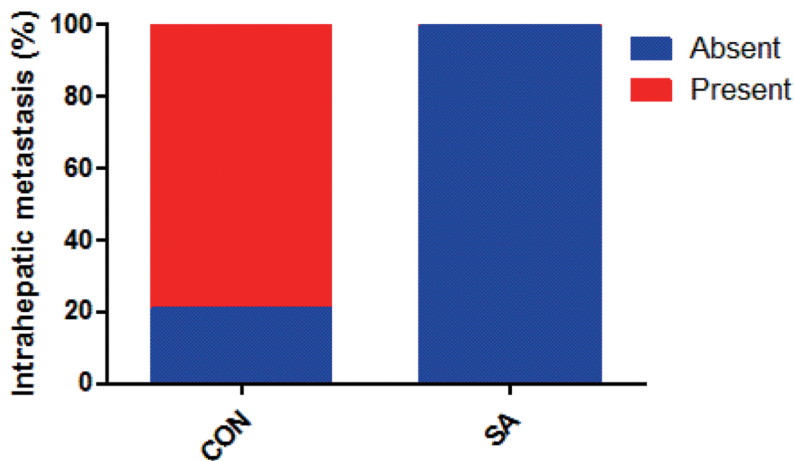

Supplement: Supplementary file 2 [file image1.pdf]

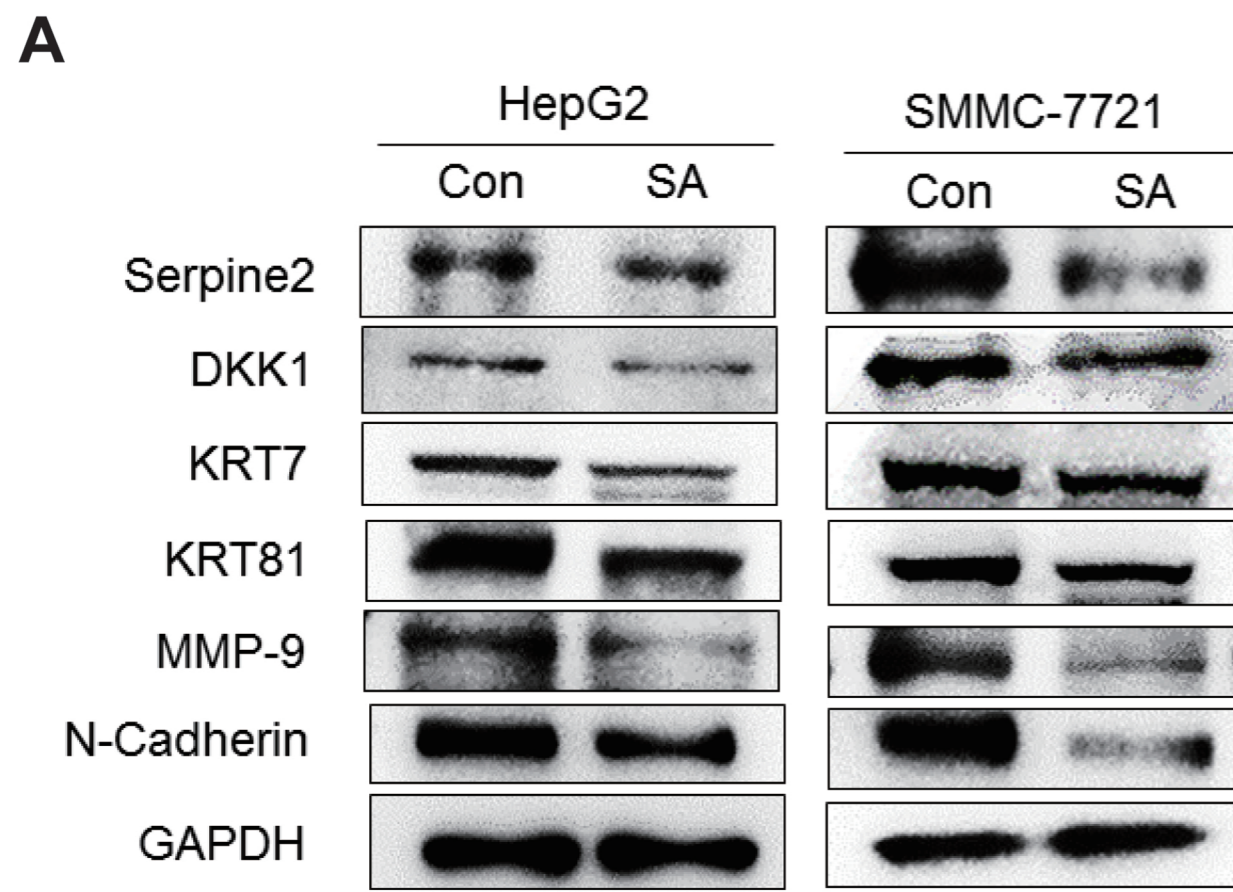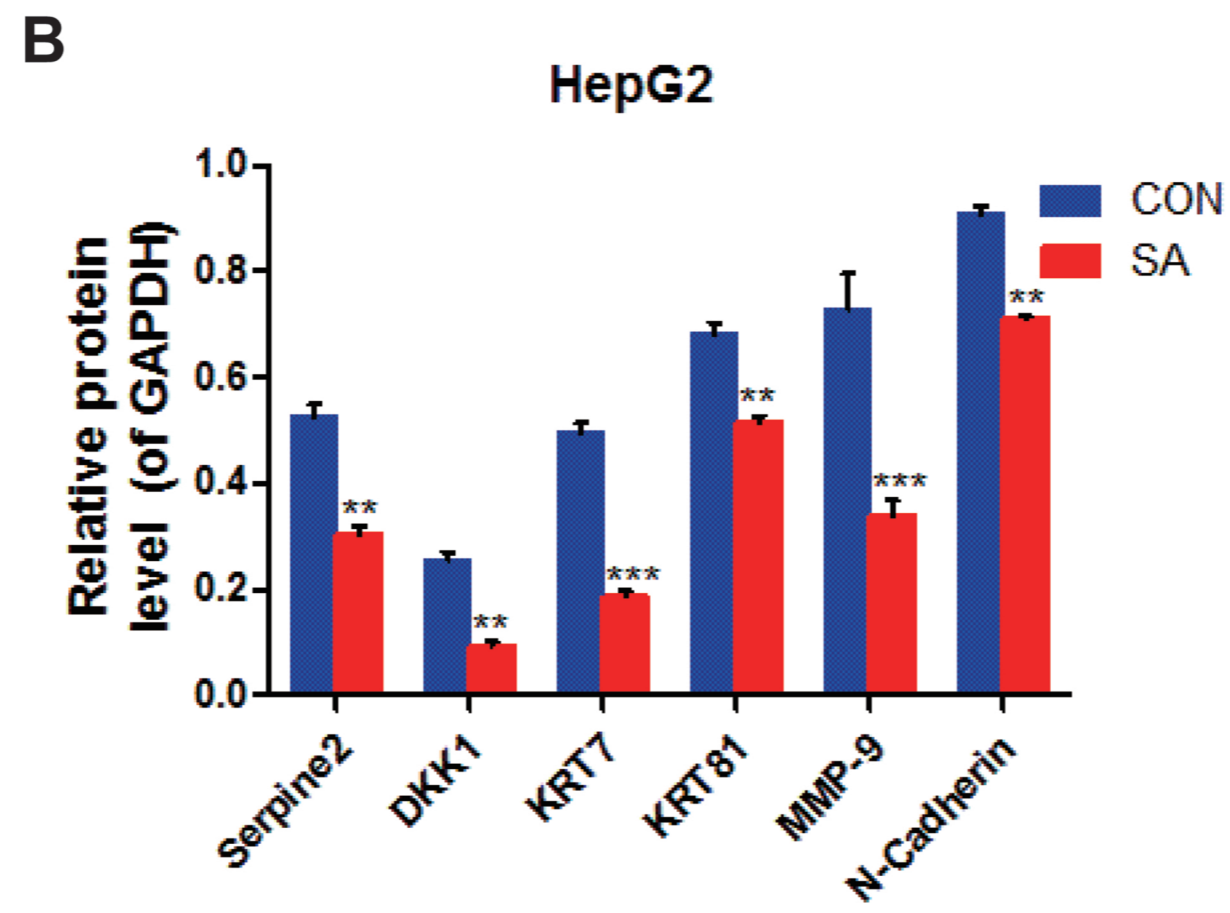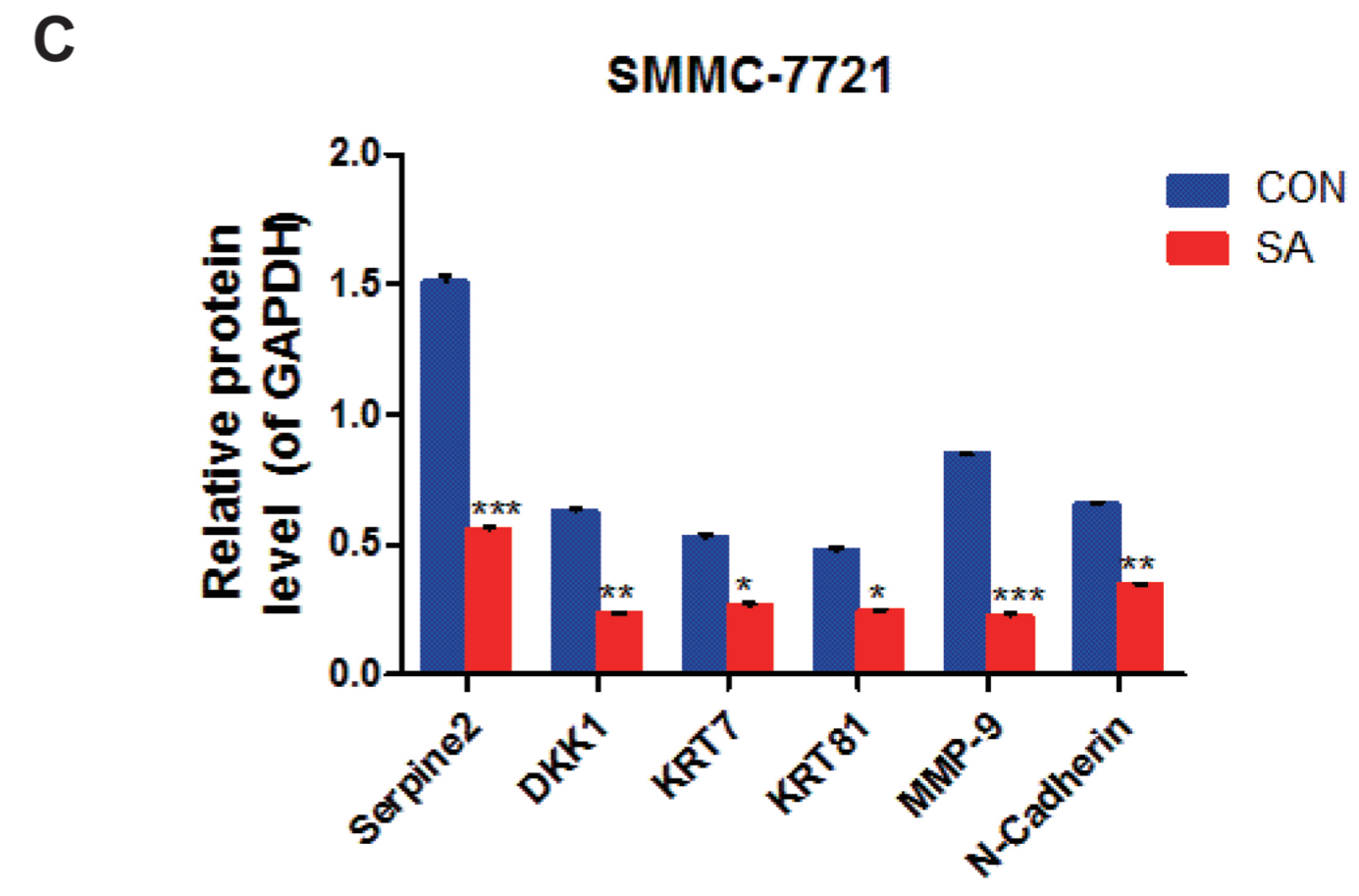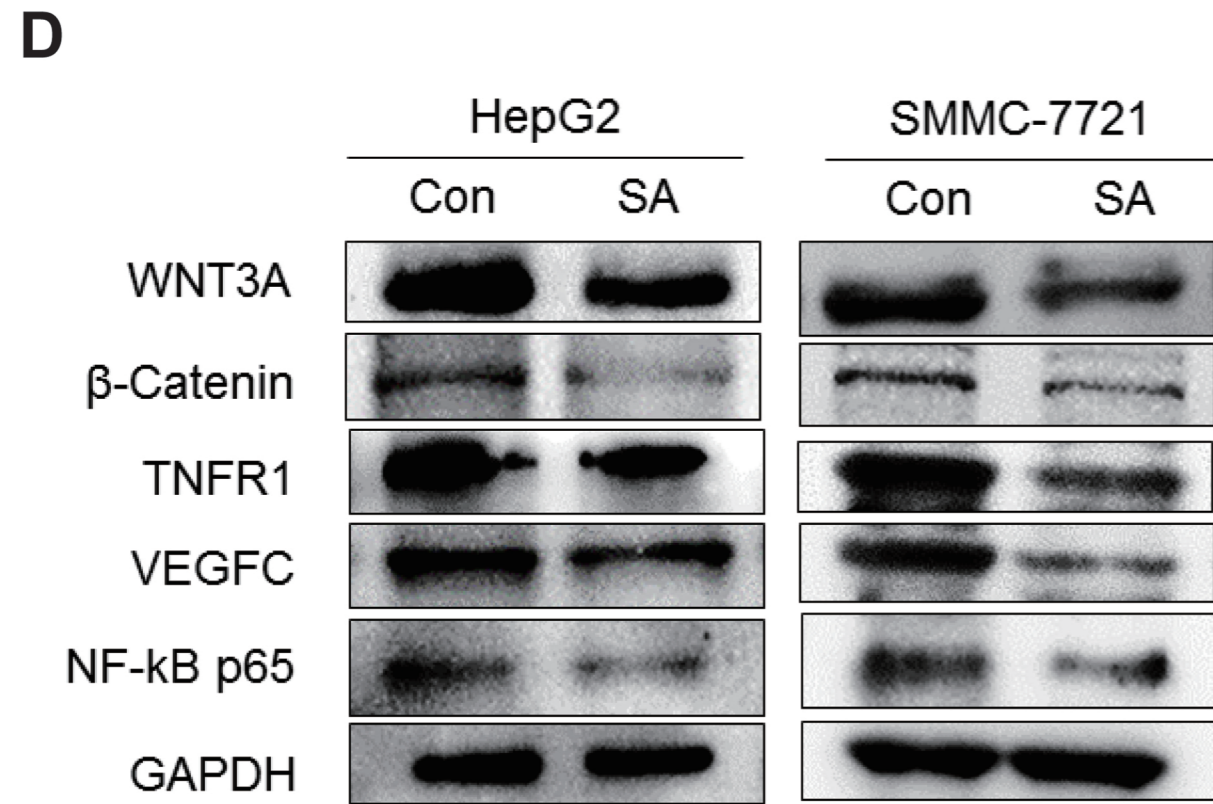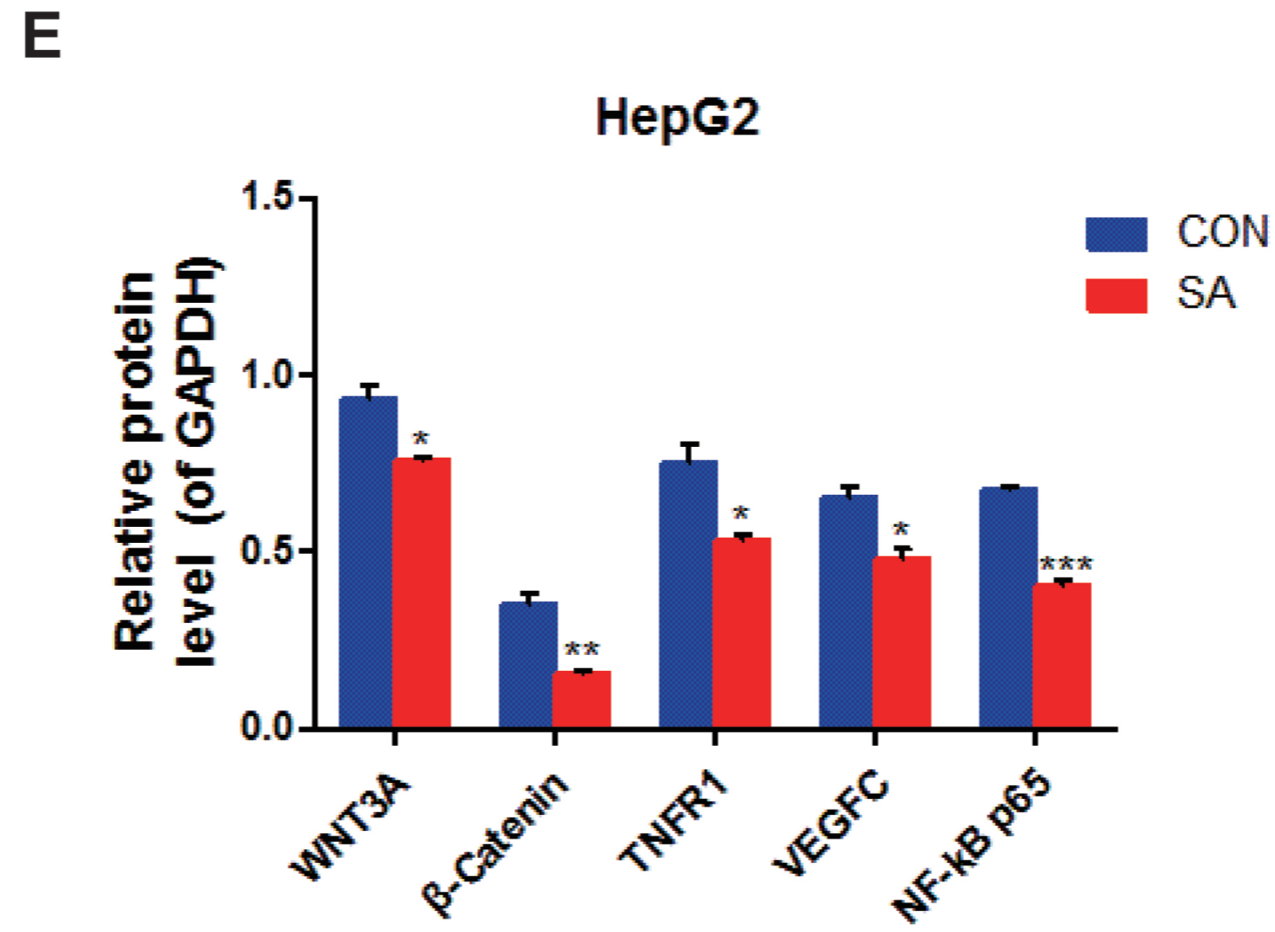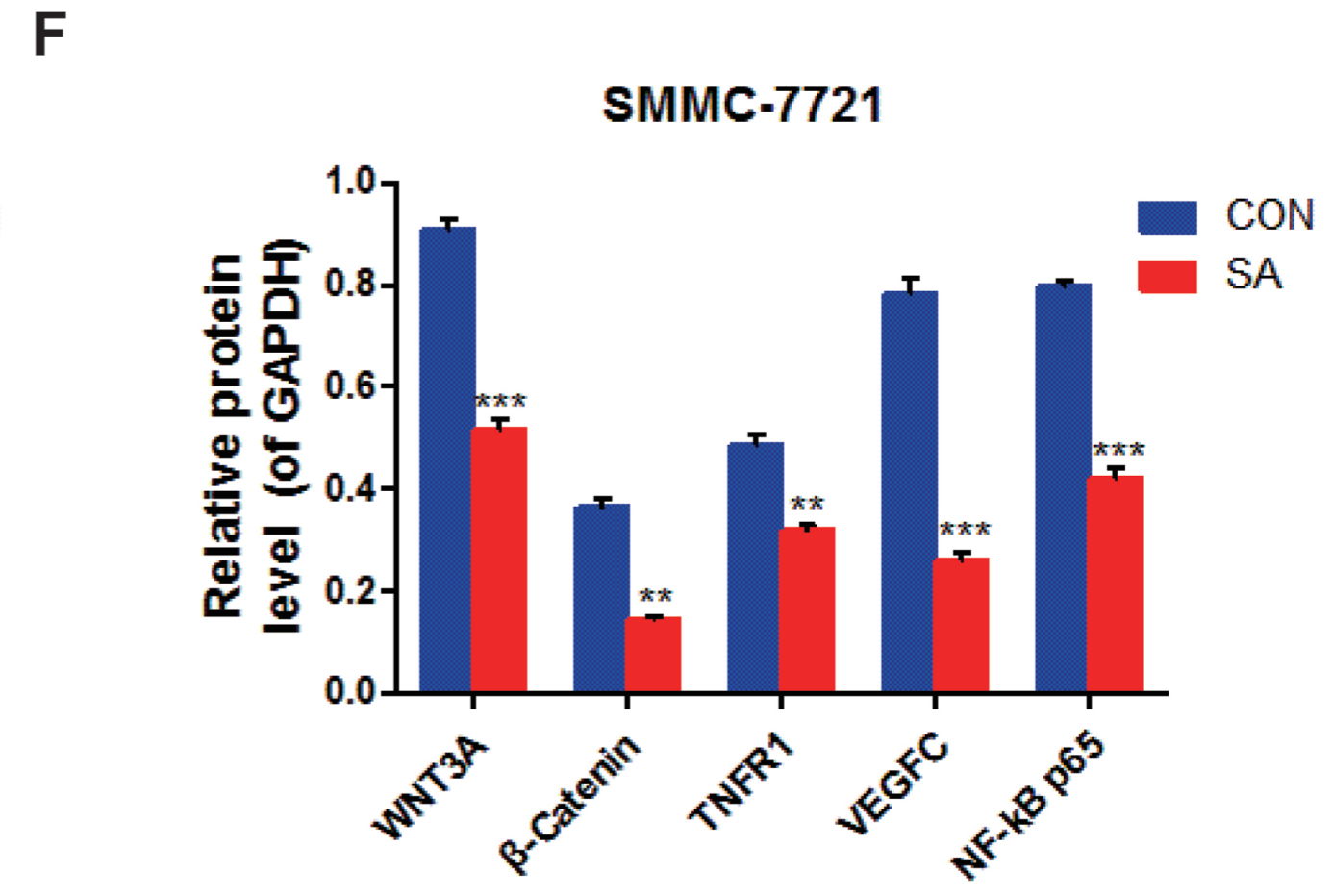

Supplement: Supplementary file 3 [file image2.pdf]

A

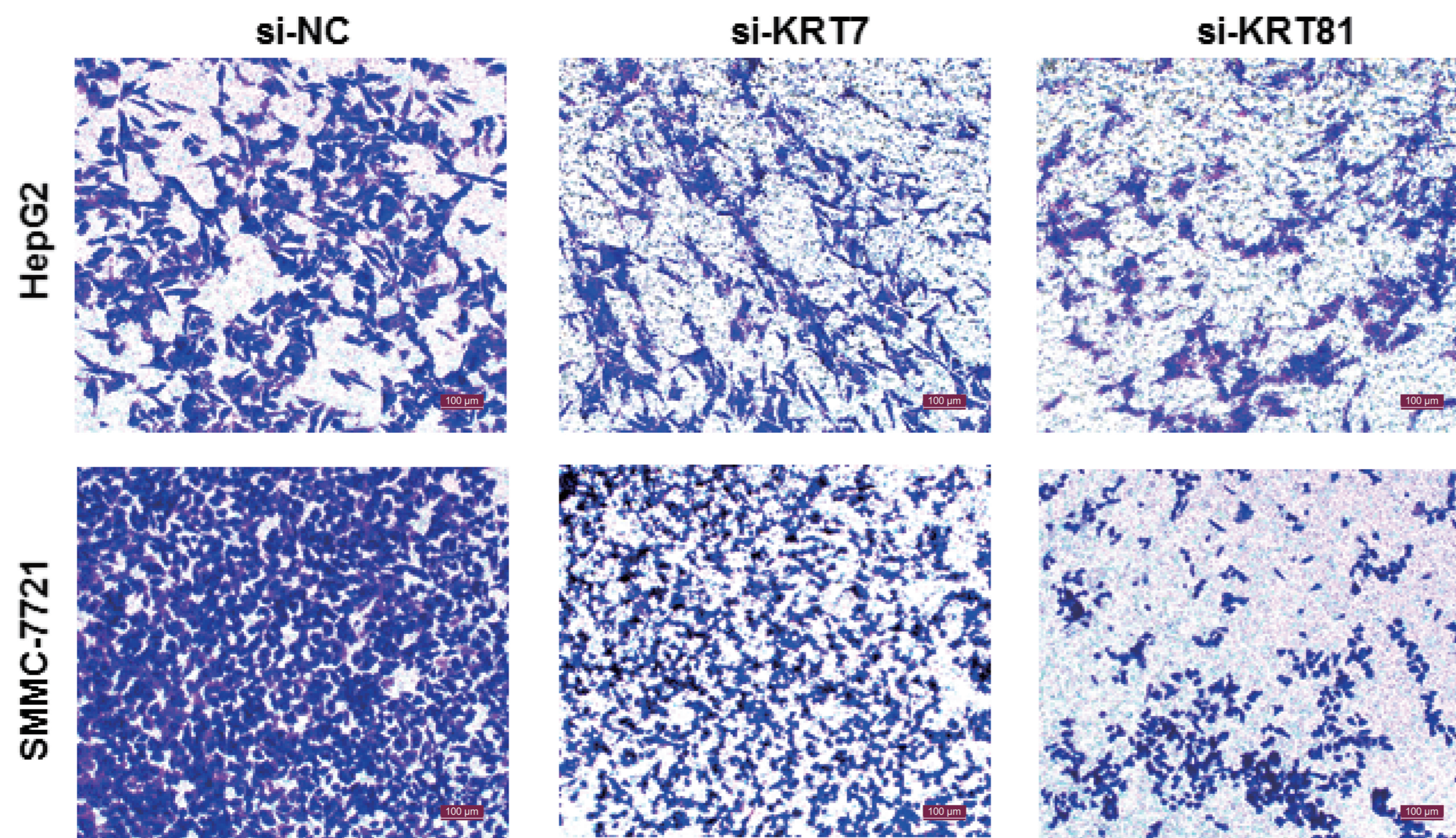

B

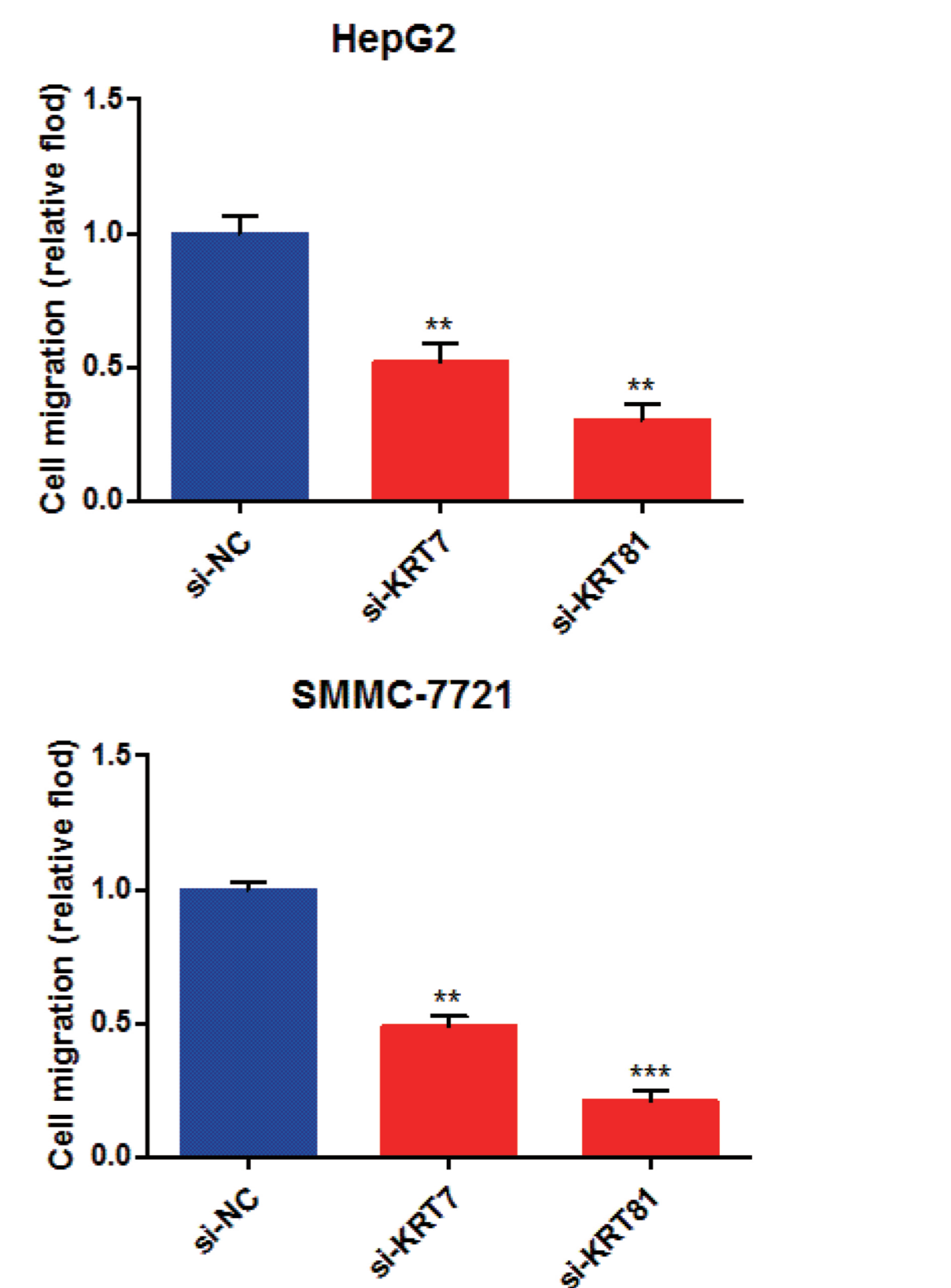

C

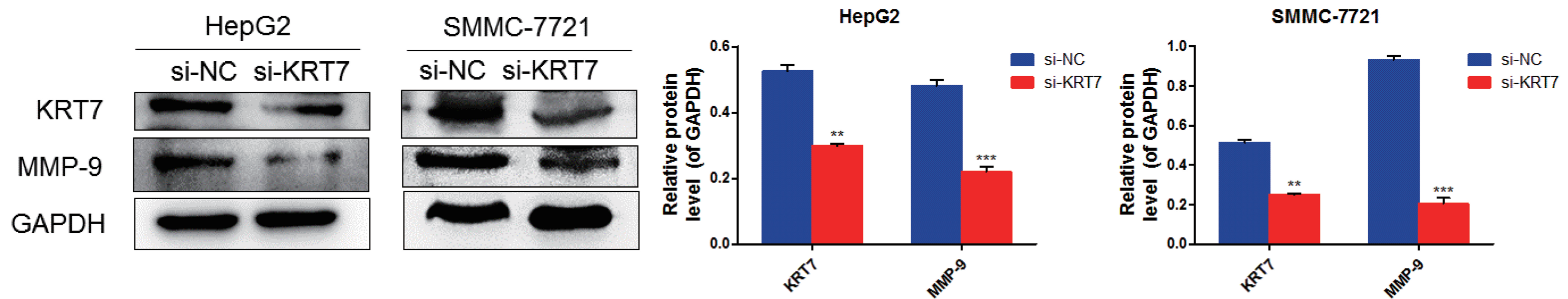

D

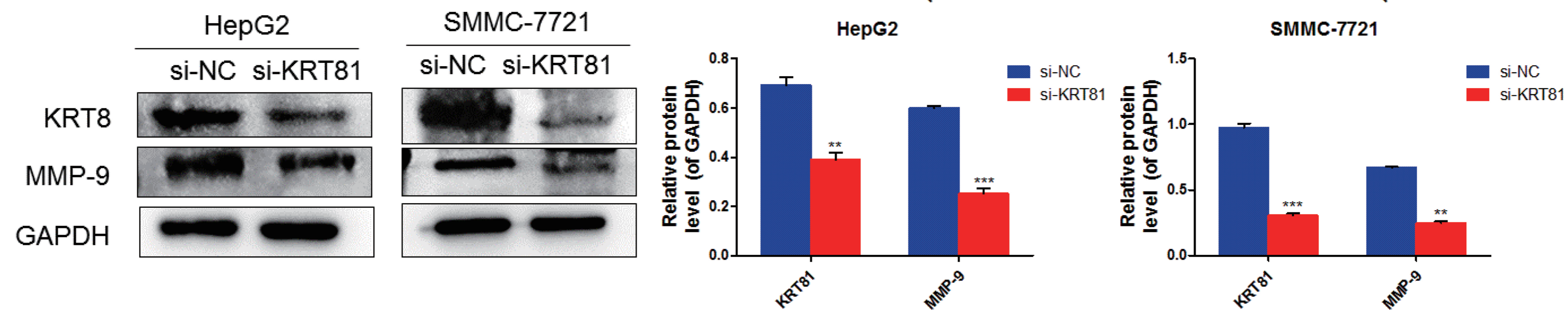

Supplement: Supplementary file 4 [file image3.pdf]
